# Methodology for non-target screening of sewage sludge using comprehensive two-dimensional gas chromatography coupled to high-resolution mass spectrometry

**DOI:** 10.1007/s00216-017-0429-0

**Published:** 2017-06-23

**Authors:** Cathrin Veenaas, Peter Haglund

**Affiliations:** Department of Chemistry, Faculty of Science and Technology, Umeå University, 90187 Umeå, Sweden

**Keywords:** Non-target screening, Sewage sludge, Method development, GC-HRMS, GC × GC

## Abstract

To investigate the wide range of pollutants occurring in sewage sludge, an analytical method for comprehensive non-target screening is needed. To the best of our knowledge, no procedures currently exist for the full screening of organic contaminants in sewage sludge, which is the ultimate goal of this project. We developed non-discriminating sample preparation methods for gas chromatography-mass spectrometry (GC-MS) analysis. Pressurized liquid extraction (PLE) was used for extraction, with in-line (silica gel selective PLE, SPLE) or off-line clean-up (gel permeation chromatography, GPC). This combination allowed the analysis of non-polar compounds of all sizes and small semi-polar and non-polar compounds. The results show that the combination of SPLE and PLE with GPC is suitable for analysis of established as well as new contaminants. Both methods were validated for 99 compounds with different properties. For all GC suitable analytes, either one of the methods produced acceptable recoveries (64 to 136%). As a test, the two methods were used for non-target screening of Swedish sewage sludge. A tiered approach was used to tentatively identify the sludge contaminants. In total, 1865 and 1593 compounds were found of which 321 and 192 compounds were tentatively identified for the PLE and SPLE method, respectively. For a comprehensive coverage of contaminants, the two methods should be used together, with the PLE method covering a wider polarity range and the SPLE method a wider size range. In addition, polar substances will require liquid chromatography-mass spectrometry analysis, the method for which will be developed soon.

**Electronic supplementary material:**

The online version of this article (doi:10.1007/s00216-017-0429-0) contains supplementary material, which is available to authorized users.

## Introduction

Globally, there are more than 100,000 chemicals currently used every day [1]. Many of these chemicals, among them potential pollutants, are disposed of in wastewater and hence enter sewage treatment plants (STPs). STPs are used to remove nutrients, but also some metals and organic chemicals, from urban water to create a less contaminated effluent. Consequently, STPs form a link between the technosphere and the environment. A by-product of the sewage treatment process is sewage sludge—a solid product that contains nutrients as well as pollutants. These nutrients make the sewage sludge attractive for applications such as fertilizer for agriculture, provided that the contaminant levels are not too high.

Statistics have shown that 54% of the sewage sludge in Europe and 61% of the North American sewage sludge are used in land applications, whereas 10 and 17%, respectively, are placed in landfills [2, 3]. The rest of the sludge is either combusted, disposed of, or reused in other ways.

In order to safely dispose of or reuse the sewage sludge in agriculture the European Union directive 86/278/EEC forces member states to monitor heavy metal concentrations in sludge and soil on a regular basis when sewage sludge is used as fertilizer [4]. Similarly, the US government has defined maximum pollutant loadings for sewage sludge when used as fertilizer on agricultural crops. Again, heavy metals were the only regulated pollutants [5]. For EU member states, national requirements apply as well. Some of these are much stricter than the EU laws (e.g., in Denmark, Finland, Sweden, and the Netherlands) and some of them also include organic contaminants in sewage sludge [6]. In Sweden, for example, maximum loadings for polychlorinated biphenyls (PCBs), nonylphenol ethoxylates (NPEs), polycyclic aromatic hydrocarbons (PAHs), and toluene are defined and those are measured before sludge is spread on crops [6]. Nevertheless, sewage sludge might still pose a risk when used on arable land as in all cases only target compounds are monitored and some crops are known to take up pollutants from soil [7–9]. This study aims to develop a method that enables a comprehensive screening of sewage sludge and thereby allowing detection and monitoring of currently unknown organic contaminants present in sewage sludge. Although the literature contains many examples that deal with the analysis of sewage sludge, no study so far has involved non-target screening of sewage sludge, which is the scope of this study. Traditionally, sludge has been extracted using Soxhlet and ultrasound extraction, but nowadays, these methods are often replaced with pressurized liquid extraction (PLE), as highlighted by Zuloaga et al. [10]. By reducing solvent consumption and process time, providing improved extraction rates, and enabling extraction of polar as well as non-polar compounds [10, 11], PLE would appear to be a suitable method for the comprehensive extraction of sewage sludge. In addition, PLE provides the opportunity of an in-cell clean-up using, for example, Florisil, silica gel, alumina [12], or combinations of them [13]. Such procedures, known as selective PLE (SPLE), decrease the amount of co-extracted interfering matrix compounds from solid samples, such as lipids or humic and fulvic acids [14] and may, therefore, enable direct analysis after extraction [13]. SPLE has been applied to various matrices such as soil and sediment [15–17], food and feed samples [18], and sewage sludge [13, 19] and dates back to 1996 when the use of alumina was suggested in a Dionex application note to retain fat [12].

In SPLE, there is a balance between the polarity range of chemicals extracted and the purity of the extracts. Polar solvents or solvent mixtures will extract a wide range of chemicals but will also extract more of the matrix. Although in some cases, only filtering and/or derivatization is required before analysis by gas chromatography-mass spectrometry (GC-MS) [13, 19], some analysts apply further clean-up prior to analysis [20, 21] in order to reduce interference and improve the limit of detection (LOD) [22]. Conventional PLE will, in most cases, require further clean-up. For a non-target screening, a non-destructive clean-up is generally used, such as gel permeation chromatography (GPC) [22], liquid-liquid partitioning, or adsorption chromatography.

The combination of PLE and GPC has previously been used for target and non-target analysis of solid matrices [23, 24]. While PLE can be employed with various different types of solvent, a binary mixture of a non-polar solvent (such as *n*-hexane) and a more polar solvent (such as dichloromethane (DCM)) is often used [25]. For example, Kim et al. [26] extracted polychlorinated dibenzo-*p*-dioxins, dibenzofurans, and biphenyls from animal feed using *n*-hexane/DCM (1:3) and Muscalu et al. [27] extracted halogenated organic compounds from soil, sediment, and sludge using *n*-hexane/DCM (3:1). However, no studies have focused on a comprehensive non-target screening of organic contaminants in sewage sludge.

The current study aimed to develop procedures for non-target screening of sewage sludge. For this purpose, PLE with off-line GPC clean-up was compared to SPLE with in-cell silica clean-up. The extraction efficiency and the amount of co-extracted matrix were assessed for several solvents and solvent mixtures. As a test of utility, the two methods were used for non-target screening of Swedish sewage sludge samples. The number of sample constituents captured and the spectra quality (percentage of peaks that could be tentatively identified) obtained with the two non-target screening techniques were compared. There was also an assessment of whether a combination of the two approaches would enlarge the chemical domain covered. Finally, the potential need for complementary LC-MS analyses was discussed.

## Materials and methods

### Experiment overview

The method development and evaluation included two methods for extraction, PLE and SPLE, two methods for solvent evaporation, Turbovap and Rotavap, and two methods for sulfur removal, using acid-activated copper and tetra butyl ammonium sulfite (TBA) reagent, respectively. In addition, a method validation study was carried out using spiked and unspiked sewage sludge.

### Materials

The 8270 MegaMix® standard (see Electronic Supplementary Material (ESM) Table S1 for compound information) was bought from Restek (Bellefonte, PA, USA). Deuterated PAHs (see ESM Table S2 for more information) were obtained from Cambridge Isotope Laboratories (Tewksbury, MA, USA). Sand (Fontainebleau PROLABO®) and 2-propanol (HiPerSolv Chromanorm 100%, PROLABO) were purchased from VWR (Leuven, Belgium), whereas concentrated hydrochloric acid was obtained from VWR, Fontenay-sous-Bois (France). Sodium sulfate, silica gel 60, acetone (≥99.8%), *n*-hexane (≥98.0%), and cyclohexane were obtained from Merck KGaA (Darmstadt, Germany). DCM (99.99% purity), isooctane (99.94% purity), ethyl acetate (99.96% purity), and methanol (99.99% purity) were purchased from Fisher Scientific (Loughborough, UK). Copper of mesh size 10–40 (≥99.9% purity) and sodium sulfite (≥98%) were acquired from Sigma-Aldrich (St. Louis, MO, USA). TBA hydrogen sulfate was purchased from Molekula (Shaftesbury, UK). Glass fiber filter papers (GFFs) with a diameter of 27 mm were acquired from Dionex (Sunnyvale, CA, USA). An Omnifit glass column (L 50 cm, i.d. 25 mm) from Diba Industries Ltd. (Cambridge, United Kingdom) and SX-3 Bio-Beads from Bio-Rad Laboratories AB (Hercules, CA, USA) were used for GPC.

### Sludge sampling and sample pre-treatment

Digested, dewatered sludge (15 days in digester) was obtained from the STP in Umeå, Sweden. Samples were taken in the morning and frozen immediately until further use. Prior to extraction, the samples were freeze-dried using a Lyovac GT 2 (SRK System Technik GmbH, Riedstadt, Germany) equipped with an Edwards High Vacuum Pump E2M2, and the dry weight was determined (~33.6%). Afterwards, the sludge was homogenized using a mortar and pestle. As a third step directly before extraction, a filling material, either pre-cleaned (PLE with acetone) sand or pre-baked (550 °C) sodium sulfate, was mixed with the dried sludge (approximately 3:2, *w*/*w*) to create a homogeneous mixture that filled the extraction cells evenly. The detailed procedures are described below.

### Extraction equipment and conditions

Sample extraction was carried out using a Dionex™ ASE™ 350 system equipped with 22-mL stainless steel extraction cells under the following conditions: 120 °C, 5 min static extraction, 3 extraction cycles, 100% flush volume, and 60 s nitrogen purge. To reduce the risk of contamination, high purity solvents were used. During the method development, different solvents and solvent combinations were tested. The solvent volume used for the extraction resulted in approximately 50 mL under the specified conditions. More information on the solvents can be found in the respective sections below. In addition, the extraction cells, sand, and GFFs were pre-cleaned using the PLE system with acetone under the following conditions: 100 °C, 1 min static extraction, 3 extraction cycles, 100% flush volume, and 60 s nitrogen purge.

### PLE method development experiments

Non-polar solvents such as *n*-hexane are expected to release less co-extracted matrix but may not exhaustively extract contaminants. Binary solvent mixtures generally offer better extraction efficiencies. The method development therefore included the following solvents and solvent mixtures: *n*-hexane, *n*-hexane/DCM (80:20, *v*/*v*), and *n*-hexane/DCM (50:50, *v*/*v*). The polar modifier selected, DCM, is aprotic and known to efficiently desorb difficult to extract compounds such as PAHs from solid matrices [28]. Moreover, conventional PLE and SPLE with silica were compared. To assess the suitability of the methods, the co-extracted matrix amount and extraction efficiency were determined and compared.

For the evaluation of the co-extracted matrix, 1-g sludge aliquots mixed with sand for homogenization were extracted as described above. After extraction, the solvent was fully evaporated and the residue was determined gravimetrically (*d* = 0.001 g). For the extraction efficiency evaluation, analytical standards were spiked to sand as follows: the PLE cells were filled with a GFF and pre-cleaned sand and spiked with approximately 1 μg of the 8270 MegaMix standard and the SPLE extraction cells were filled with a GFF, 5 g silica gel 60 (dried at 130 °C for 12 h or overnight), a second GFF on top, and pre-cleaned sand and spiked with approximately 1 µg of the 8270 MegaMix standard. A blank containing one GFF and sand and one GFF, silica gel 60, another GFF, and sand were prepared for the PLE and SPLE method, respectively. After solvent exchange to isooctane and volume reduction to about 1 mL, d10-phenanthrene (approximately 544 ng per sample) was added as the volumetric standard. Analysis was carried out using an Agilent 7890A GC (Agilent Technologies, St. Clara, CA, USA) coupled to a high-resolution (HR) time-of-flight (TOF) MS (HRT; Leco Corp. St. Joseph, MI, USA) with electron impact (EI) ionization. The instrument was equipped with a Gerstel CIS4 inlet, which was operated in pulsed splitless mode. The splitless time was 105 s with an inlet purge flow of 25 mL/min and septum purge flow of 3 mL/min. A 30-m DB-5MS Ultra Inert column (0.25 mm i.d., 0.25 μm film thickness) from Agilent was used. The oven program was as follows: 80 °C (3.8 min), 15 °C/min, and 300 °C (6.5 min). Helium was used as carrier gas with a flow of 1 mL/min. The transfer line was held at 300 °C. The ion source temperature was 250 °C and 12 spectra per second were recorded in the range from *m*/*z* 38 to 400.

A calibration curve with ten points ranging from 1 to 1000 ng/mL was prepared. A linear regression curve with a fixed intercept at zero was used for the determination of the analyte concentrations.

### Solvent evaporation experiments

The MegaMix standard was used for testing two methods of solvent evaporation. The first method used a Turbovap concentration workstation (Biotage AB, Uppsala, Sweden), operated at 35 °C and 500 mbar, and the second method used a rotary evaporator (Rotavap) from Heidolph (Schwabach, Germany), operated at 50 °C with a pressure below 150 mbar. The Turbovap was used with 60 mL PLE vials and the Rotavap with 100 mL pear-shaped flasks, which were tilted to create a horizontal solvent surface and minimize the deposition of chemical residues on dry walls. A MegaMix aliquot equivalent of 1 μg of each analyte was added to 50 mL isooctane, which was then evaporated to 1 mL using the two techniques. The experiments were carried out in triplicate and a blank containing only solvent was included. A mix of deuterated PAHs was added as a volumetric standard and the samples were analyzed with GC-MS, as described in the “Method development” section.

### PLE method validation experiments

A total of 99 analytes, including the MegaMix 8270, PCBs, organophosphates, fragrances, pesticides, and others, were used for validation of the final method. For information about native standards/analytes and labeled standards including their spiking levels, please refer to the ESM Tables S1, S3, and S4. The added amounts of native analytes were higher than of labeled standards in order to sufficiently exceed the intrinsic sludge levels. Three sets of samples were prepared in triplicate for each method: (i) 1 g sewage sludge spiked with native and labeled compounds, (ii) sewage sludge spiked with labeled compounds, and (iii) inert material (pre-baked sodium sulfate) spiked with labeled compounds (blanks).

Each set was extracted using PLE and SPLE with *n*-hexane/DCM (80:20, *v*/*v*), leading to a total of 24 samples. Samples extracted with PLE (not SPLE) were further cleaned by using GPC (mobile phase, cyclohexane/ethyl acetate (3:1); flow, 5 mL/min; fraction, 23–59 min). The combination of cyclohexane and ethyl acetate is commonly used in GPC [29]. The flow was adjusted not to exceed the maximum column pressure while the collection window was determined by injecting the MegaMix and collecting fractions for subsequent GC analysis to determine when the compounds elute. The column was packed in-house with approximately 45 g SX-3 Bio-Beads and was compressed to a bed height of 40 cm. For all samples and blanks, sulfur was removed using TBA sulfite reagent as explained below. The analysis was carried out using the GC-HRT system described above, equipped with a secondary oven and a quad jet two stage thermal (liquid nitrogen) modulator for GC × GC analysis. The first column was a 30-m Rtx-5MS (0.25 mm i.d., 0.25 μm film thickness), and the second column was a 1.1 m Rxi-17Sil MS column (0.25 mm i.d., 0.25 μm film thickness), both from Restek. The oven programs were as follows: 90 °C (2 min), 5 °C/min, and 300 °C (5 min) for the first oven and 105 °C (2 min), 5 °C/min, and 300 °C (8 min) for the second. The modulator had a temperature offset of 15 °C relative to the secondary oven, and the modulation period was 4 s with a hot jet and cold jet duration of 1.2 and 0.8 s, respectively. The transfer line was held at 325 °C. The ion source temperature was 250 °C, and 150 spectra per second were recorded in the range from *m*/*z* 38 to 1000.

A six-point calibration curve was prepared of which a linear regression curve (intercept at zero) was created for the quantification of the analytes. Information about the linear range and *R* ^2^ can be found in Table S5 (see ESM). Before calculating the recovery, the amount of analyte detected in the unspiked sewage sludge, if present, was subtracted from the amount detected in the spiked sewage sludge.

### Sulfur removal experiments

Sulfur removal using activated copper and a TBA sulfite reagent were compared using triplicate treatments for recovery of the following contaminants (all at 1 ng/μL in isooctane): the 8270 MegaMix, an organochlorine pesticide mix (GC multiresidue pesticide standard no. 2), and an organophosphorus pesticide mix (GC multiresidue pesticide standard no. 8) from Restek and diazinon, 2-(methylthio) benzothiazole, and thiabendazole from Dr. Ehrenstorfer GmbH (Augsburg, Germany). The recovery of each analyte was determined using GC × GC-MS, as described in the “Method validation” section.

For sulfur removal with copper, the copper was activated using concentrated hydrochloric acid and then rinsed each three times with Milli-Q water (Merck Millipore), methanol, and DCM. The activated copper was added in small portions (~½ teaspoon) to the samples until freshly added copper no longer discolored. Samples were kept overnight in the fridge and more copper was added if additional discoloring was visible the next day.

For sulfur removal using the TBA sulfite, a reagent mixture was prepared and used as described by Jensen et al. [30]. In brief, TBA sulfite reagent was prepared by mixing 1.695 g TBA hydrogen sulfate with 50 mL Milli-Q water followed by threefold extraction, each with 15 mL *n*-hexane for removal of impurities. Afterwards, the solution was saturated with 12.5 g sodium sulfite. Samples in 2 mL isooctane were mixed with 1 mL 2-propanol and 1 mL TBA sulfite reagent. The mixture was shaken and sodium sulfite was added in 100-mg portions until a solid residue remained after shaking. Then, 5 mL Milli-Q water was added and the mixture was shaken for another minute. Afterwards, the mixture was centrifuged (10 min, 2000 rpm) and the supernatant was transferred.

### Data evaluation

The limit of quantification (LOQ) and LOD were derived from method validation blank values (see section “PLE method validation experiments”) where possible. In all other cases, they were determined using the standard deviation of the triplicate injections of the lowest point of the standard curve. The formulae for the LOD and LOQ are as follows:

$$ \mathrm{LOD}=3.3\times \frac{\sigma}{S} $$

$$ \mathrm{LOQ}=10\times \frac{\sigma}{S} $$

where *σ* is the standard deviation of the response (blank or standard dilution close to the LOQ, respectively) and *S* being the slope of the standard curve.

The peak finding and library search for the non-target application were carried out using the ChromaTOF software (version 1.90.60) from Leco Corporation in connection with the NIST MS library (2011). For a peak to be accepted, the following criteria had to be fulfilled: (i) the area of the peak in the sample had to be at least three times higher than the area of the same peak in the blank and (ii) the peak had to be found in at least two out of three sample replicates. The stepwise procedure of identifying and classifying peaks in sludge chromatograms was as follows:

1. Peaks occurring in the blank (in high enough concentrations) as well as the sample were removed (as defined above).
2. Features that occurred only in one of the triplicates were removed.
3. Peaks were classified into groups according to the rules in Table 1 in combination with the regions defined in Table 1 and Fig. 2 (see “Results” section). All classification regions followed the upwards trend (increasing second dimension retention time) caused through the isothermal (starting at 41 min) in the end of the oven temperature program.
4. The remaining peaks were identified using the NIST library (similarity and probability), fragmentation patterns, and, where possible, retention indexes. Only hits with a similarity match greater than 500 were displayed. To reduce the amount of peaks to look at, only peaks that had a first hit with either a high similarity (>750) or a high probability (>7000) were considered. For compounds where no retention index was found, a simple linear regression model using retention times of standard analytes and their boiling points was used for giving an approximate retention time. Retention times were used for exclusion purposes rather than confirmation.
5. Chlorine and bromine filters were applied. Firstly, ChromaTOF’s built-in chlorine and bromine filters were used. In addition, our own filter criteria were applied (Table 1).
6. The mass defect was used to identify chlorinated and brominated compounds using ^81^Br–^79^Br and ^37^Cl–^35^Cl (nominal isotope spacing divided by exact isotope spacing), respectively, as reference for normalization. The mass spectrum was summed over a range of 10 min each. Since the raw chromatograms/spectra were used and peaks were identified manually, peaks that were missed in the peak picking process during the data processing could also be identified.

**Table 1:** Rules that were used for classifying peaks

Compound class	Position of region	Rules
Siloxanes	2.25–2.75 s	Abundance of *m*/*z* 73.047 ± 0.001 is ≥80% abundance of base mass AND Abundance of *m*/*z* 147.065 ± 0.001 is ≥80% abundance of base mass
Phthalates	See Fig. 2	*m*/*z* 149.023 ± 0.001 is the base mass
Long chain amides	See Fig. 2	*m*/*z* 59.037 ± 0.001 is the base mass
Long chain ketones	2.75–3.27 s (10 min) 2.75–3.51 s (41 min)^a^	*m*/*z* 58.041 ± 0.001 is the base mass
Long chain aldehydes	2.75–3.27 s (10 min) 2.75–3.51 s (41 min)^a^	Abundance of *m*/*z* 57.070 ± 0.001 is ≥75% abundance of base mass AND Mass *m*/*z* 41.039 ± 0.001 is present
Alkanes	See Fig. 2	Abundance of *m*/*z* 57.070 ± 0.001 is ≥90% abundance of base mass AND Abundance of *m*/*z* 71.086 ± 0.001 is ≥10% abundance of base mass AND Abundance of *m*/*z* 43.055 ± 0.001 is ≥10% abundance of base mass
Alkenes	See Fig. 2	Abundance of *m*/*z* 55.054 ± 0.001 is ≥75% abundance of base mass
Fatty acids	See Fig. 2	Abundance of *m*/*z* 60.021 ± 0.001 is ≥60% abundance of base mass AND Abundance of *m*/*z* 73.029 ± 0.001 is ≥75% abundance of base mass
Chlorinated compounds	No region defined	Loss of Cl_2_ from the molecular ion OR Loss of Cl from the molecular ion OR Loss of HCl from the molecular ion OR Loss of Cl and gain of H from the molecular ion OR Abundance of CCl (*m*/*z* 46.968 ± 0.001)
Brominated compounds	No region defined	Loss of Br_2_ from the molecular ion OR Loss of Br from the molecular ion OR Loss of HBr from the molecular ion OR Loss of Br and gain of H from the molecular ion

The corresponding regions for the range between 10 and 41 min are given in the table, where possible, or shown in Fig. 2 in the “Results” section

^a^The classification regions are becoming broader towards the end of the run

The in silico fragmentation tool MetFrag [31] was used to identify unknown chlorinated compounds (steps 5 and 6 above). MetFrag uses compound structures stored in databases (e.g., PubChem or Chemspider) to predict the fragmentation of small molecules. Those fragmentation patterns are then compared to a spectrum that is inserted by the user. The similarity of the spectrum inserted by the user to the predicted fragmentation is then given. Originally, MetFrag was developed for tandem MS data but, it can also be applied for EI MS data.

Here, the internet database Chemspider was used as a source for candidate structures matching the neutral mass of the highest *m*/*z* present in the spectrum, with a 5 ppm mass tolerance. The electron ionization spectra for the unknown compounds were exported from ChromaTOF and compared to the fragments generated by MetFrag from [M+] using a 5 ppm or 0.001 mDa tolerance. Only compounds including (at least) carbon, hydrogen, and chlorine were considered. The Chemspider data source count and reference count were taken into account in scoring the results. Hereby, the spectral match was weighted with 100%, while the data source count and reference count were weighted with 50% each.

## Results

### Method development

During the method development, PLE and SPLE extraction efficiencies were compared for different solvents or solvent mixtures. For both methods, there was an improvement in extraction efficiency when changing from *n*-hexane as the pure extraction solvent to the 20% DCM in *n*-hexane mixture but almost no improvement when increasing the DCM percentage to 50%. For PLE and SPLE, the median recovery percentages for the 20% DCM in *n*-hexane mixture were almost identical at 71 and 76%, respectively. The 10-percentile values did, however, differ greatly, with greater than 10-fold higher recovery values for PLE (48%) than for SPLE (3%), which is no surprise as SPLE is a more selective extraction technique. For both methods, early eluting compounds showed lower extraction efficiencies or recoveries than later eluting compounds. An evaluation of evaporation methods was, therefore, carried out prior to the validation study.

The amount of co-extracted matrix increased with the amount of DCM used in the extraction solvent mixture and was higher for PLE than SPLE in all cases. The percentages of co-extracted material using *n*-hexane, 20% DCM in *n*-hexane, and 50% DCM in *n*-hexane were 5, 6, and 7% for PLE and 0.7, 1.6, and 2.5% for SPLE, respectively. As the extraction efficiency was considerably higher for the 20% DCM mixture than for pure *n*-hexane, without showing a significant increase for the 50% DCM mixture, the less polar solvent mixture was chosen for both PLE and SPLE. This mixture also releases slightly less matrix compared to the more polar mixture.

### Solvent evaporation

**Fig. 1:**
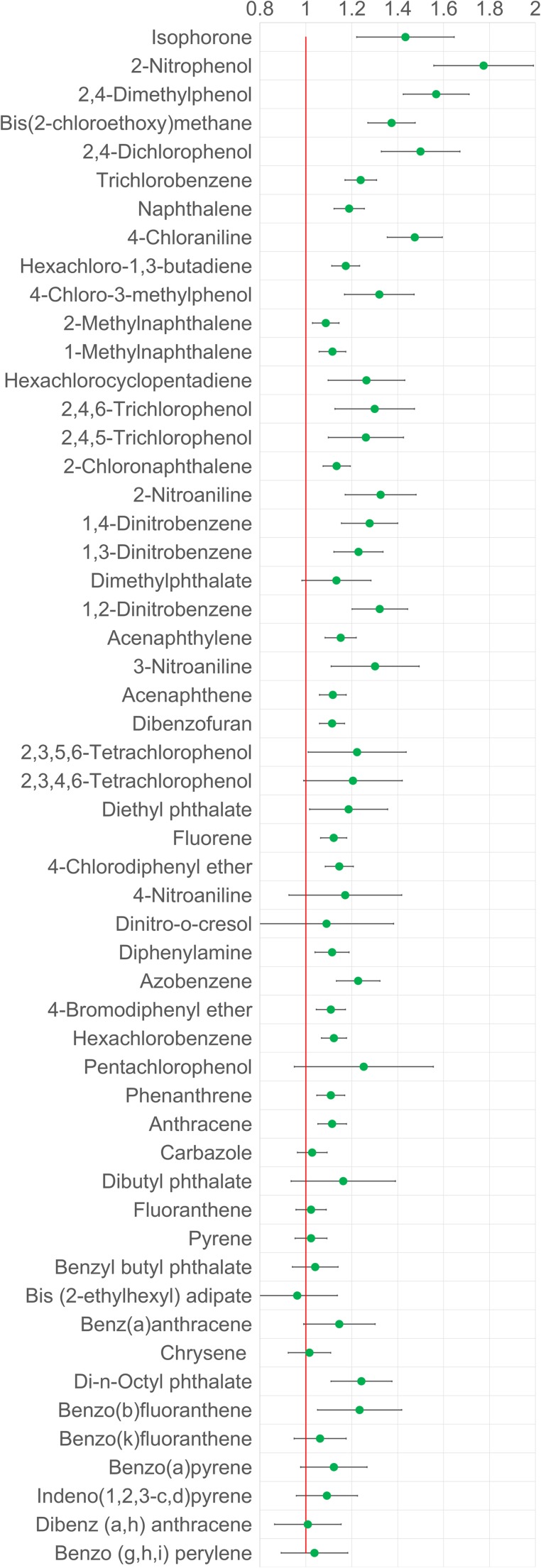
Compound recovery for evaporation: ratio of Rotavap to Turbovap. The ratio of the averages (*n* = 3) and the total error resulting from error propagation are shown and compounds are sorted by retention time, i.e., boiling point. 4-Nitrophenol was excluded from the dataset due to very high standard deviations for both methods

The graph comparing Rotavap versus Turbovap evaporation for solvent volume reduction (Fig. 1) clearly shows that the ratio is above one predominantly and, thus, that Rotavap gives a better result, i.e., higher recovery of analytes. Only bis(2-ethylhexyl) adipate showed a slightly better recovery using Turbovap. However, the difference is not significant. As expected, the difference between the methods was relatively small for high molecular weight analytes such as large PAHs and larger for low molecular weight analytes such as mono- and di-substituted benzenes, phenols, and anilines. On average, the recoveries can be improved by approximately 20% by using Rotavap instead of Turbovap. Rotary evaporation was therefore used during the method validation.

### Method validation

The method validation included two different ways of extraction and clean-up, PLE followed by GPC, and SPLE with silica as the in-cell clean-up sorbent. Both methods used 20% DCM in *n*-hexane as the extraction solvent. The two methods were able to extract most of the analytes. However, some analytes had to be excluded from the dataset due to poor GC performance (see Table 2, footnote). A few other compounds (2,4-dimethylphenol, 2,4-dichlorophenol, bisphenol A) also suffered from relatively poor chromatography and their results are, therefore, slightly more uncertain, as illustrated by a relatively high variation among replicates. Those data were still kept in the dataset for comparison purposes.

**Table 2:** Average recovery values (*n* = 3) obtained during the method validation for the PLE method (PLE followed by GPC) and the SPLE method (selective PLE using silica as sorbent)

Compound^a^	Recovery PLE method (%) ± StDev	Recovery SPLE method (%) ± StDev	Compound	Recovery PLE method (%) ± StDev	Recovery SPLE method (%) ± StDev
PAHs			Phthalates, phosphates		
Naphthalene	135 ± 74	123 ± 54			
Acenaphthylene	136 ± 24	146 ± 12	Dimethyl phthalate	101 ± 16	<LOQ
Acenaphthene	109 ± 33	90 ± 29	Diethyl phthalate	88 ± 31	2 ± 1
Dibenzofuran	110 ± 26	128 ± 28	Dibutyl phthalate	43 ± 15	4 ± 12
Fluorene	109 ± 32	110 ± 35	Benzyl butyl phthalate	85 ± 6	<LOQ
Phenanthrene	78 ± 39	121 ± 24			
Anthracene	110 ± 29	154 ± 7			
Fluoranthene	95 ± 7	94 ± 32	Tributylphosphate	99 ± 19	<LOQ
Pyrene	70 ± 6	68 ± 79	TCEP	21 ± 2	<LOQ
Benz(*a*)anthracene	123 ± 22	185 ± 6	TDCPP	57 ± 2	<LOQ
Chrysene	79 ± 3	143 ± 3	Triphenylphosphate	59 ± 2	<LOQ
Benzo(*b*+*k*)fluoranthene	69 ± 3	104 ± 1	TBEP	59 ± 13	<LOQ
Benzo(*a*)pyrene	<LOQ	82 ± 87	EHDPP	51 ± 11	<LOQ
Indeno(1,2,3-*c*,*d*)pyrene	<LOQ	68 ± 16	Triethylhexylphosphate	3 ± 5	<LOQ
Dibenz(*a*,*h*)anthracene	<LOQ	73 ± 8			
Benzo(*g*,*h*,*i*)perylene	<LOQ	80 ± 16			
PCBs			Phenolics		
PCB 81	111 ± 12	95 ± 24	2,4-Dimethylphenol	211 ± 138	56 ± 43
PCB 77	112 ± 28	143 ± 30	2,4-Dichlorophenol	177 ± 126	37 ± 40
PCB 123	130 ± 4	130 ± 26	Bisphenol A	198 ± 100	<LOQ
PCB 118	137 ± 6	128 ± 10	4-Chloro-3-methylphenol	111 ± 24	<LOQ
PCB 114	130 ± 4	107 ± 24	2,4,6-Trichlorophenol	48 ± 2	287 ± 136
PCB 105	147 ± 12	107 ± 23	2,4,5-Trichlorophenol	110 ± 3	186 ± 172
PCB 167	116 ± 2	108 ± 13	2,3,5,6-Tetrachlorophenol	36 ± 5	<LOQ
PCB 156	115 ± 4	121 ± 23	2,3,4,6-Tetrachlorophenol	49 ± 3	<LOQ
PCB 157	119 ± 3	117 ± 11	Pentachlorophenol	101 ± 10	<LOQ
PCB 189	123 ± 9	122 ± 16			
Diphenyl ethers			Pesticides, pharma		
4-Chlorodiphenyl ether	106 ± 36	106 ± 24	Diazinon	73 ± 2	<LOQ
4-Bromodiphenyl ether	98 ± 11	99 ± 26	Chlorpyrifos	81 ± 9	43 ± 15
BDE-28	99 ± 3	126 ± 15	Dacthal	92 ± 3	96 ± 7
BDE-47	107 ± 3	129 ± 62	*p*,*p*′-DDE	72 ± 7	133 ± 23
BDE-99	87 ± 10	110 ± 8	Carbamazepine	95 ± 3	<LOQ
BDE-154	132 ± 21	123 ± 1	Triclosan	78 ± 12	104 ± 5
BDE-153	122 ± 59	<LOQ			
Other non-polar compounds			N compounds		
Trichlorobenzene	135 ± 64	111 ± 53	2,6-Dinitrotoluene	134 ± 15	101 ± 9
Hexachlorobenzene	111 ± 3	108 ± 2	1,2-Dinitrobenzene	28 ± 2	11 ± 2
Bis(2-chloroethoxy)methane	91 ± 71	61 ± 55	Azobenzene	133 ± 38	137 ± 45
Hexachloro-1,3-butadiene	127 ± 54	110 ± 39	Diphenylamine	117 ± 34	135 ± 51
Octachlorostyrene	114 ± 2	111 ± 6			
Fragrances					
Isophorone	103 ± 78	<LOQ			
Galaxolide	30 ± 28	108 ± 61			
Tonalide	90 ± 28	145 ± 19			
Musk xylene	65 ± 8	96 ± 36			
Musk ketone	137 ± 22	49 ± 30			

*TCEP* tris(2-chloroethyl) phosphate, *TDCPP* tris(1,3-dichloropropyl) phosphate, *TBEP* tris(2-butoxy-ethyl) phosphate, *EHDPP* 2-ethylhexyldiphenyl phosphate

^a^3- and 4-Nitroaniline, 4-chloraniline, 2- and 4-nitrophenol, 2,4-dinitrophenol, dinitro-*o*-cresol and hexachlorocyclo-pentadiene were removed from the dataset due to poor GC performance, and PCB 169 and dioctyl phthalate were removed due to discrepancies among the replicates

In general, the PLE method worked better for most compounds (Table 2), although there were problems with analyzing a few large PAHs (likely due to the relatively narrow (too short) collection windows used in the GPC). The SPLE also worked well for many compounds. It does, however, show the expected losses of polar analytes, such as the organophosphates (OPs), several phenolic compounds, some phthalates, diazinon, and carbamazepine due to the sorption to the silica sorbent.

Taking both methods into account, the non-polar and moderately polar compounds (left half of Table 2 and pesticides and fragrances) showed recoveries ranging from 64 to 136%, while the LOD values ranged from as low as 0.02 ng/g for bis(2-chloroethoxy) methane to 76 ng/g for benzo(*g*,*h*,*i*)perylene (data from ESM Table S5). Several of the more polar compounds (right half of Table 2) did, however, show relatively low recoveries, including many OPs, some chlorophenols, and 1,2-dinitrobenzene. These, and other more polar compounds, would be better analyzed using a complementary LC-MS method.

### Sulfur removal

The data for the validation study (Table 2) were produced using sulfur removal with a TBA sulfite reagent, much because it has been claimed to be a soft method [30, 32]. This method was, however, found to be difficult to work with and the process time-consuming. An alternative technique using copper was therefore tested to improve the method further.

The recoveries of roughly 100 compounds were determined for the two sulfur removal techniques: copper and TBA sulfite reagent. Diethyl phthalate, bis(2-ethylhexyl) phthalate, and bis(2-ethylhexyl) adipate showed high blank values for both procedures and were therefore excluded from the dataset. In addition, endrine ketone was excluded from the dataset due to poor reproducibility, i.e., it had a high standard deviation for both procedures. The recovery values for the remaining compounds using the copper treatment ranged from 42 to 114%, while TBA sulfite reagent treatment resulted in recoveries from 38 to 127%. Median values were 90 and 85% for the copper and TBA sulfite reagent treatments, respectively. The 10-percentile and 90-percentile were calculated as 79 and 98% for copper and 53 and 98% for TBA sulfite reagent, respectively. Thus, the copper treatment was found to be slightly better with both a higher median recovery and a narrower recovery range. The copper treatment was also found to be much more user-friendly and therefore more preferable.

### Application

**Fig. 2:**
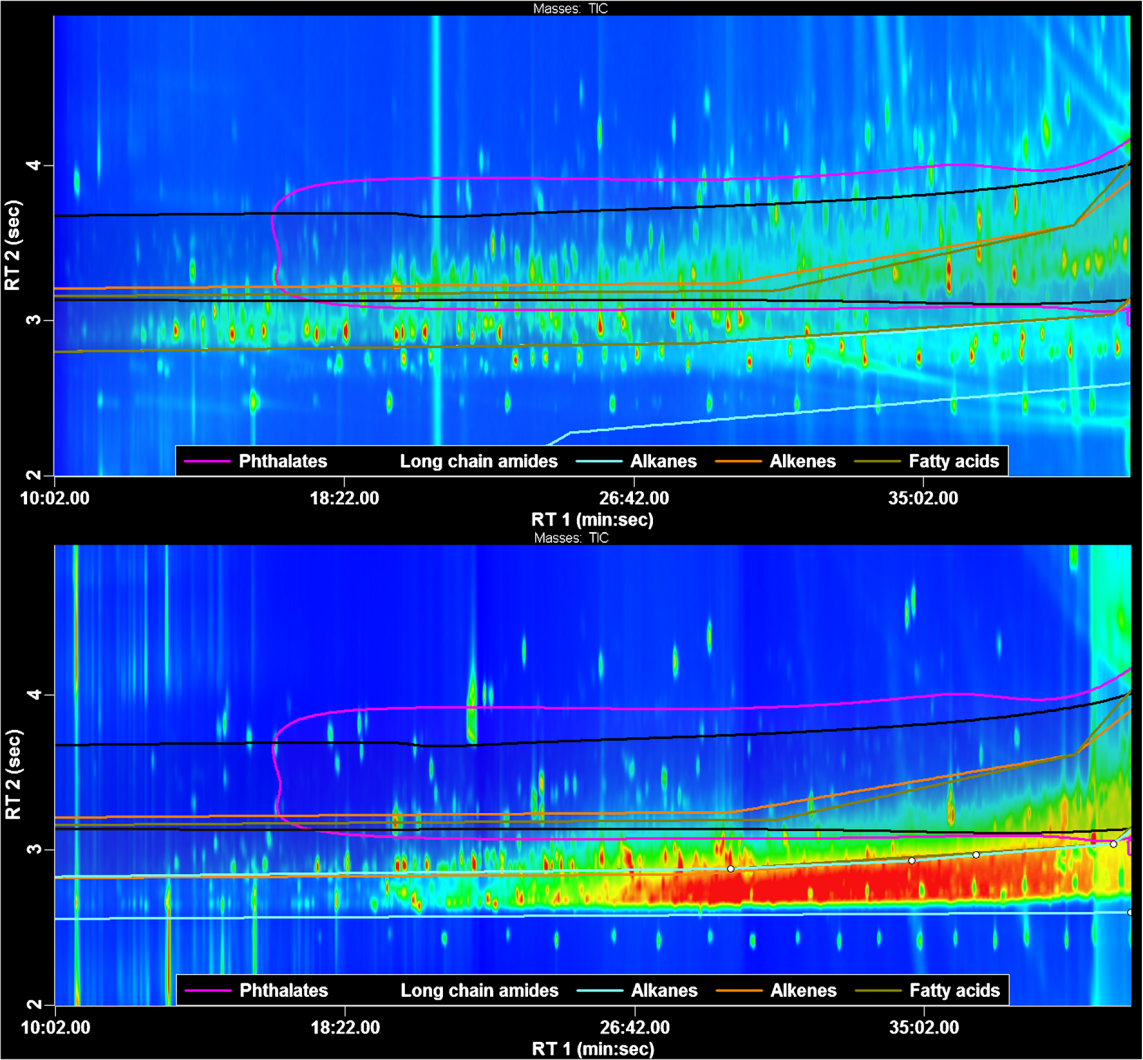
Chromatograms of a sewage sludge extract using the PLE (*top*) and SPLE (*bottom*) method. Some of the classification regions are indicated in the plot. All regions followed an upwards trend at the end of the run caused through the constant temperature set at the end of the oven program (isothermal). The second dimension scale has been offset by 2 s to enhance the presentation

In total, after removing the blanks and peaks that occurred in only one replicate, 1865 and 1593 peaks were obtained from the ChromaTOF peak finding algorithm, using sewage sludge processed with the PLE and SPLE methods, respectively. Some 633 (34%) and 378 (19%) peaks had a spectral match with a similarity equal to, or higher than, 75% for the PLE and SPLE methods, respectively, using the NIST library. Figure 2 shows the complexity of the PLE and SPLE sample extracts, and Table 3 shows how many compounds could be identified or characterized using the different techniques. Since a tiered approach was used, compounds that were identified or characterized at an early stage do not appear in later stages. Therefore, the number of identified compounds generally decreases in the later stages. In total, 174 and 45 compounds were uniquely identified (tentatively) using the PLE and SPLE method, respectively. In addition, 147 compounds were tentatively identified through both methods.

**Table 3:** Classification and tentative identification of compounds during different stages of the tiered approach

Tier	Technique	PLE	SPLE
1	Classification	231	187
2	NIST similarity	267	174
3	NIST probability	37	11
4	Chlorine/bromine filters	11	2
5	Mass defect	6	5
	Sum	552	379
	Tentatively identified	321	192

**Fig. 3:**
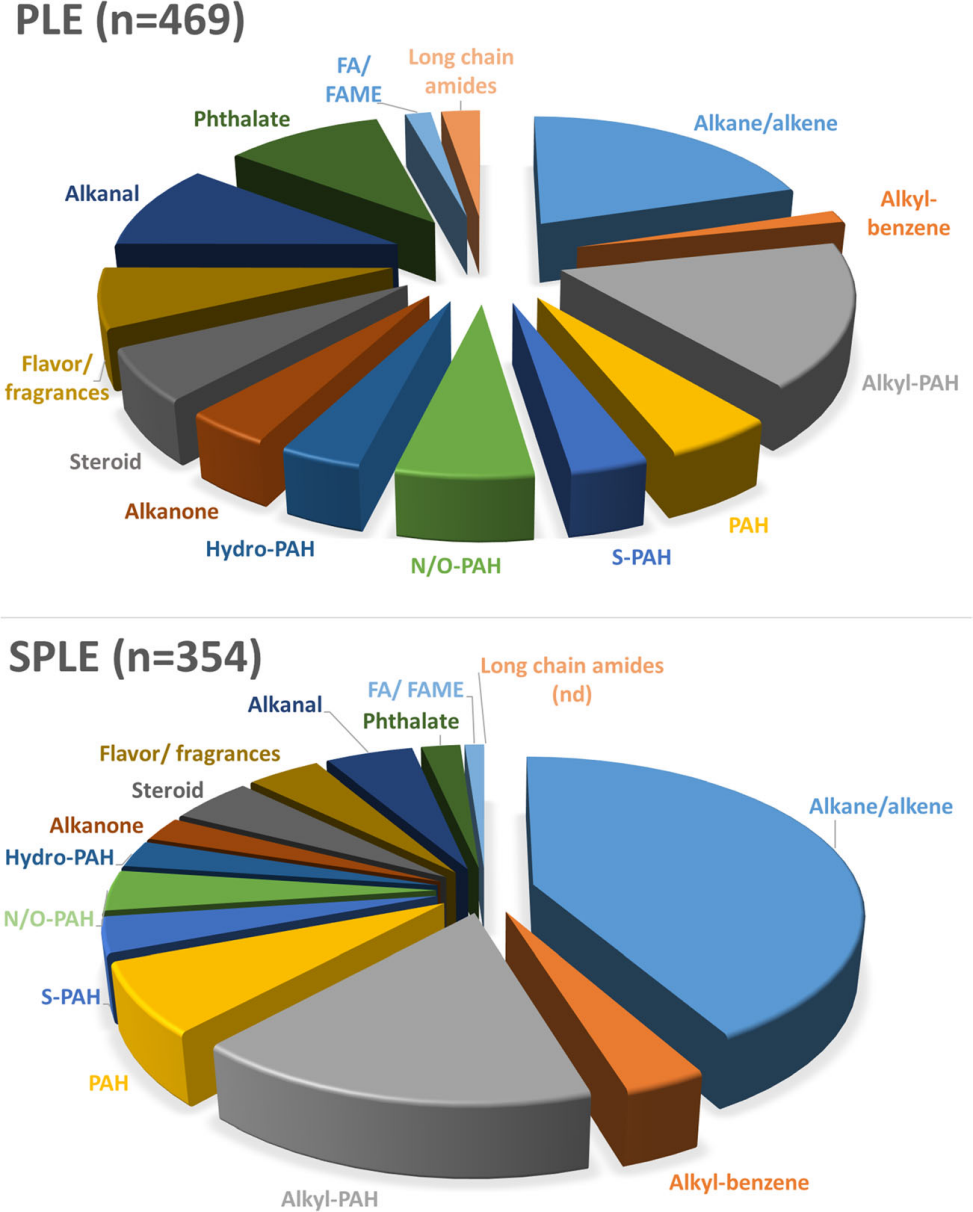
Pie charts showing the number of automatically classified compounds (through classification regions and rules; tier 1) and other grouped compounds: alkyl-benzenes, polycyclic aromatic compounds, steroids, and flavor and fragrance compounds (tiers 2 and 3) for the PLE and SPLE method, respectively. *nd* not detected, *n* total number of classified and grouped compounds depicted in the chart

The automatic peak classification detected a large number of alkanes, alkenes, and related compounds such as long-chain ketones, aldehydes, amides, as well as free fatty acids and methyl derivatives thereof. It also detected a large number of phthalates. Figure 3 shows the distribution of the classified compounds, as well as four groups of compounds, alkyl-benzenes, flavor and fragrances, PAHs and derivatives, and steroids, which contain constituents with high structural similarity that are difficult to identify correctly without using reference materials. More information on the alkyl-benzenes, flavor and fragrances (mainly terpenoid and musk compounds), PAHs and derivatives, and steroids can be found in the ESM (Table S6).

Roughly 15% of the compounds detected (17% for PLE and 12% for SPLE) could be assigned CAS numbers. A list, including tentatively identified compounds that were found in the final extracts from the PLE and SPLE methods (tiers 2 and 3), is shown in Table 4. This table also contains the first dimension retention times, the second dimension retention times, the nominal molecular weights, the mass deviation (ppm) from the theoretical molecular ion or major fragment ion mass, and the score and probability produced from reverse searches of the NIST library. The compounds identified were loosely divided into the following groups: alkyl-phenols, extractives (of plant origin), organophosphate esters, pharmaceuticals and personal care products (PPCP), stabilizers (antioxidants and UV absorbers), as well as other halogenated compounds and process chemicals.

**Table 4:** Tentatively identified compounds (tiers 2 and 3) detected in the final extracts from the PLE or SPLE methods but not included among the classified or grouped compounds (Fig. 3)

Name	PLE	SPLE	RT 1 (s)	RT 2 (s)	Exact mass (amu)	Mass dev. (ppm)	Rev. Sim.	Probability
Alkyl-phenols								
2,3,6-Trimethylphenol	X		598	3.35	136.0888	−1.20	843	1004
4-*tert*-Octylphenol	X		1102	3.26	206.1671	0.61	891	3945
4-(1-Phenylethyl)phenol	X		1258	3.78	198.1045	0.07	925	1797
4-(1,1-Dimethylhexyl)phenol	X		1274	3.26	206.1671	−3.63^a^	800	519
3-(2-Phenylethyl)phenol	X		1394	3.85	198.1045	0.87	780	8308
Extractives								
10,18-Bisnorabieta-5,7,9(10),11,13-pentaene	X	X	1706	3.58	238.1722	0.16	816	2730
Dehydroabietal	X		1890	3.73	284.2140	0.94	646	7658
Ferruginol	X		1946	3.67	286.2297	−0.18	807	8032
Methyl dehydroabietate	X	X	1958	3.62	314.2246	0.22	884	9326
4-Epidehydroabietol	X		1986	3.82	286.2297	0.59	886	8826
Dehydroabietic acid	X		2054	3.83	300.2089	0.18	871	6626
Organophosphate esters								
Tris(1,3-dichloroisopropyl)phosphate (TCPP)	X		1950	3.74	427.8839	0.79^a^	800	3712
Triphenyl phosphate (TPP)	X		2018	4.21	326.0708	−1.27	892	9785
2-Ethylhexyl diphenyl phosphate (EHDPP)	X		2038	3.61	362.1647	0.04	819	1808
Cresyl diphenyl phosphate (CDPP, 2 isomers)	X		2098	4.17	340.0864	−1.29	776	3952
Isopropyl-phenyl diphenyl phosphate (iPrDPP)	X		2142	3.99	368.1177	0.30	844	9782
Dicresyl phenyl phosphate (DCPP, 2 isomers)	X		2174	4.13	354.1021	−0.53	754	9590
Tricresyl phosphate (TCP, 3 isomers)	X		2246	4.16	368.1177	−0.38	766	4497
PPCP								
2-(Dodecyloxy)ethanol	X		1246	2.96	230.2246	−1.46^a^	852	2165
Diphenylmethoxy acetic acid	X		1422	4.11	242.0943	−2.85^a^	906	3682
Clorophene	X		1482	3.90	218.0498	1.64	541	1107
1-Dodecyl-2-pyrrolidinone	X		1726	3.34	253.2406	−1.84	–^b^	–^b^
Bromhexidine	X		2030	3.85	373.9993	7.56	654	9097
Phenyl tetradecyl carbonate	X	X	2090	3.28	334.2508	−1.02^a^	805	488
2-Palmitoylglycerol	X		2106	3.28	330.2770	−2.16^a^	757	6467
Dronabinol	X		2126	3.71	314.2246	−0.23	837	8158
Cannabinol	X		2190	3.78	310.1933	0.91	828	8959
Clozapine	X		2494	5.08	326.1298	1.80	751	9637
Stabilizers, antioxidants								
1-(4-*tert*-Butylphenyl)propan-2-one	X		878	3.37	190.1358	−0.24	770	9179
Butylated hydroxytoluene (BHT)	X	X	970	3.09	220.1827	0.32	753	4398
*tert*-Octyldiphenylamine	X	X	1894	3.60	281.2143	0.68	883	9053
4,4′-Di-*tert*-butyl-diphenylamine	X	X	1914	3.53	281.2143	1.50	841	9243
2,6-Bis(1-phenylethyl)phenol	X		2042	4.11	302.1671	0.81	733	7948
2,4-Bis(1-phenylethyl)phenol	X		2098	4.10	302.1671	0.30	811	9590
*N*,*N*′-Diphenyl-1,4-benzenediamine	X		2270	4.78	260.1313	0.46	811	8932
4-Octyl-*N*-(4-octylphenyl)benzenamine	X	X	2486	3.73	393.3396	−1.02	755	4325
Vitamin E γ		X	2570	3.77	416.3654	1.11	890	4711
Vitamin E α		X	2630	3.97	430.3811	−1.53	881	3036
Vitamin E α acetate		X	2678	4.02	472.3916	0.08	890	5930
Stabilizers/screens, UV								
Benzophenone	X		1138	3.85	182.0732	0.28	937	8596
2-Ethylhexyl salicylate	X		1362	3.14	250.1569	0.81	938	9088
Phenyl cinnamonitrile	X		1454	4.06	205.0891	−2.34	751	4638
Homosalate	X		1462	3.23	262.1569	1.19	893	9078
Oxybenzone	X		1630	3.99	228.0786	−3.02	787	9504
Tinuvin P	X		1670	3.90	225.0902	0.34	874	5428
2-Ethylhexyl *trans*-4-methoxycinnamate	X		1930	3.44	290.1882	1.47	825	5485
Tinuvin 326	X		2154	3.70	315.1138	0.86	775	9757
Octocrylene	X		2258	3.76	361.2042	−0.43^a^	841	9505
Other halogenated compounds								
2,3-Dichlorobenzenamine	X		702	3.71	160.9799	0.78	728	2295
4-Chloro-m-xylenol	X		782	3.46	156.0342	0.68	842	5857
2,3,4-Trichlorobenzenamine	X	X	1014	3.81	194.9409	0.22	857	4321
4-Iodophenylacetonitrile	X		1298	4.45	242.9545	0.38	873	7929
*p*,*p*′-DDD	X	X	1814	3.87	317.9537	0.54	891	4180
6,7-Dichloro-4b,10-ethenobenz(*a*)azulene	X		1898	0.01	272.0160	1.27	668	7628
*trans*-Permethrin	X		2282	3.89	390.0789	−3.77^a^	761	7312
Other process chemicals								
*m*-Aminophenylacetylene		X	638	3.38	117.0578	−0.22	813	1506
2,3,6,7-Tetramethylquinoxaline	X		1194	3.68	186.1157	−0.10	858	9541
2,4-Diphenyl-4-methyl-1-pentene	X	X	1346	3.46	236.1565	0.41	912	4963
2,4-Diphenyl-4-methyl-2(*E*)-pentene	X	X	1398	3.43	236.1565	0.28	896	8164
4-Methoxydibenzyl	X		1438	3.78	212.1201	1.21	870	904
Hexadecanenitrile	X		1470	3.04	237.2456	−3.48^a^	792	3542
Diphenyl sulfone		X	1518	4.43	218.0402	0.19	872	9326
2-Mercaptobenzothiazole	X		1558	4.79	166.9863	0.71	702	8161
4-Stilbenol	X		1646	4.01	196.0888	−0.31	855	3608
Isopropylthioxanthone (ITX)	X	X	1998	4.22	254.0765	−0.03	805	9093
4-Benzoylbiphenyl	X		2070	4.31	258.1045	−0.52	867	7924
2,4-Bis(2-phenylpropan-2-yl)phenol		X	2114	3.92	330.1984	−0.55	874	9718

CAS numbers and IUPAC names are listed in Table S7 in the supplementary material

^a^Mass deviation was calculated from a fragment ion.

^b^1-Decyl-2-pyrrolidinone is in the NIST library. The spectral match was good; however, the retention time did not match. 1-Dodecyl-2-pyrrolidinone showed a good retention time match but has no corresponding spectrum in the NIST library. Hence, no similarity and probability values are given

Further searches for halogenated chemicals using halogen-specific filters (tier 4) or mass defect plots (tier 5) revealed 17 additional chlorinated compounds, 11 using Cl/Br filters and six using mass defect plots (Table 5). All chemicals that could be tentatively identified were aromatic compounds. Five compounds could be tentatively identified using MetFrag. The final searches, using mass defect filters, captured only chlorinated biphenyls.

**Table 5:** Halogenated compounds detected using halogen filters (tier 4) or mass defect plots (tier 5) on the final extracts of the PLE or SPLE methods

Name	PLE	SPLE	RT 1 (s)	RT 2 (s)	Exp. MW (amu)	Mass dev. (ppm)	Detection technique
Dichloroxylenol^b^	X		798	3.42	189.9952	1.54	Cl/Br filter
PCB 92	X	X	1682	3.72	323.8834	0.31^a^	Mass defect
DDMS (DDT metabolite)	X	X	1702	3.79	283.9926	6.77	Cl/Br filter
PCB 101	X	X	1730	3.63	323.8834	−2.74^a^	Mass defect
Triphenylchloromethane	X	X	1762	3.93	278.0862	0.78	Cl/Br filter
9,10-Di(chloromethyl)-9,10- dihydroanthracene	X		1806	3.85	276.0473	0.86	Cl/Br filter
PCB 151	X		1838	3.69	357.8444	−4.24	Mass defect
PCB 149	X	X	1866	3.74	357.8444	1.63	Mass defect
PCB 153	X	X	1918	3.52	357.8444	−2.84	Mass defect
Methoxy or hydroxyl, methyl-dichloro-phenanthrene/anthracene	X		1926	4.27	276.0101	0.39	Cl/Br filter
PCB 138	X	X	1978	3.83	357.8444	−6.04	Mass defect
4-(3,4-Dichlorophenyl)tetralone^b^	X		2042	4.33	290.0262	−0.79	Cl/Br filter
Dichloroflavone or dichlorophenylcoumarin^b^	X		2118	4.47	289.9894	0.64	Cl/Br filter
Dichloroflavone or dichlorophenylcoumarin^b^	X		2150	4.48	289.9897	−0.39	Cl/Br filter
Isomer of 4-(3,4-dichlorophenyl)tetralone^b^	X		2170	4.42	290.0257	0.94	Cl/Br filter
9,10-Di(chloromethyl)anthracene	X		2374	4.84	274.0316	1.44	Cl/Br filter
*p*-(6-Chloro-4-phenyl-2-quinolyl)aniline	X		2578	5.73	330.0924	−0.52	Cl/Br filter

Only tentative structures/formulae are given. CAS numbers and IUPAC names (if applicable) are listed in Table S7 in the supplementary material

^a^Mass deviation was calculated from a fragment ion

^b^These spectra for these compounds were processed using MetFrag and the compounds were identified as a result thereof

## Discussion

### Method development

In SPLE with silica, the polar sorbent retains polar compounds such as phenols, leading to very low recoveries. Non-polar compounds such as PAHs show no difference in extraction efficiency between PLE and SPLE since they are not affected by the silica. As mentioned before, the amount of co-extracted matrix increases with increasing percentage of DCM. DCM is more polar than *n*-hexane and, for this reason, it has the ability to elute more polar compounds but also more matrix compounds, e.g., humic and fulvic acids from the sewage sludge. The challenge is to find the proper balance between analyte extraction and matrix retention. For sewage sludge, 20% DCM in *n*-hexane seems to be the best compromise.

**Fig. 4:**
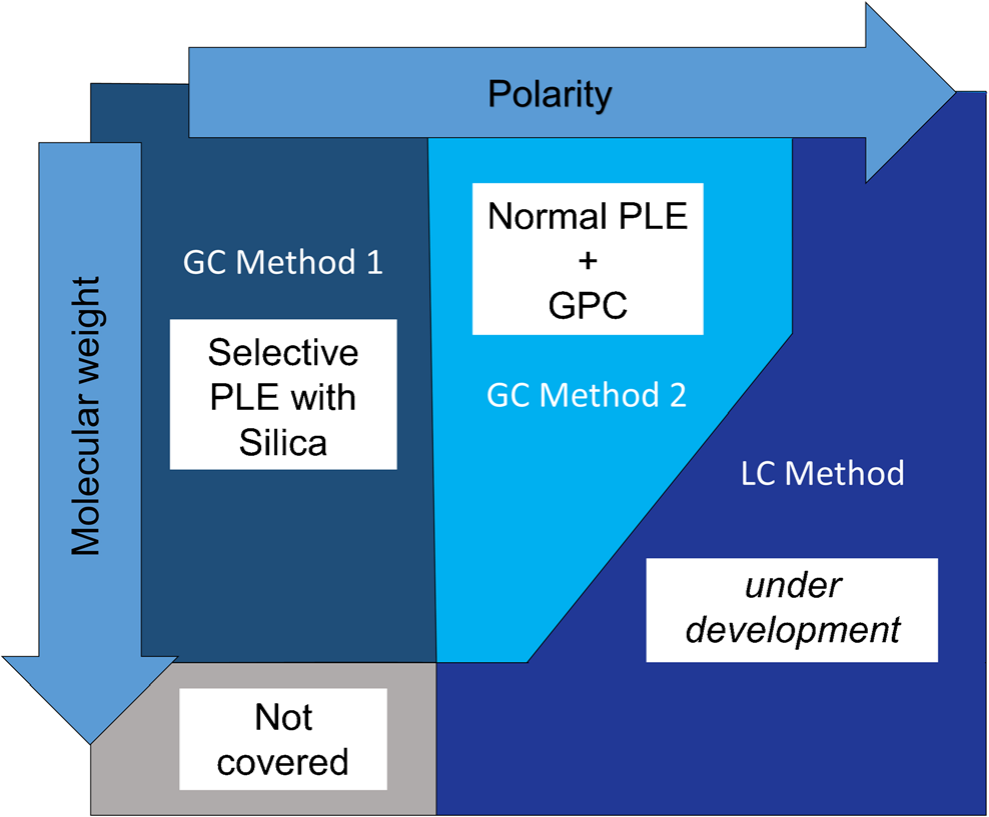
Proposed methods in relation to compound properties

In the PLE method, the clean-up was carried out using size exclusion since common matrix compounds such as lipids and humic and fulvic acids are big molecules. However, there is also a risk of losing other large GC-amenable compounds. The combination of both methods will allow a comprehensive screening of GC-amenable analytes for non-target screening of sewage sludge. The PLE method allows detection of compounds that are non-polar or moderately polar, but rather small, while the SPLE method allows the detection of relatively non-polar compounds of all sizes. The more polar analytes would have to be analyzed by LC-MS, which is currently being evaluated. Thus, using three complementary methods, a highly comprehensive non-target screening of environmentally relevant organic contaminants in sewage sludge and similar matrices (e.g., soil and sediment) could be achieved, see Fig. 4. Admittedly, very large (molecular weight of 2000 and above) non-polar contaminants would still not be possible to analyze by the three proposed methods (Fig. 4), but those are generally not bioavailable (i.e., too large to pass biological membranes).

### Solvent evaporation

During the method development, it was realized that the extraction efficiency for the studied analytes correlated with the retention time. Early eluting compounds show, in general, lower extraction efficiencies compared to later eluting compounds. Since a non-polar column was used here, the elution order is determined by the boiling point of the analytes. Early eluting compounds have a lower boiling point, while late eluting compounds have a higher one. Thus, the boiling point of the analytes seems to correlate with the recovery of the PLE and SPLE method. There is a possibility that compounds with a lower boiling point were lost in the subsequent evaporation step, which was therefore further evaluated.

The results of the comparison of Rotavap and Turbovap clearly show that Rotavap is the technique of choice for solvent evaporation for the tested compounds. The advantage of Rotavap is that the walls of the small pear-shaped flasks used in this study are constantly covered with a film of solvent. This prevents analytes from transferring to the gas phase by evaporation or adsorbing strongly to the glass walls. In the Turbovap on the other hand, a constant flow of air creates a vortex that speeds up the evaporation but also leads to the formation of a film of dry sample on the vial walls, which may cause a loss of analyte by evaporation or adsorption. Hence, greater losses and lower recoveries are to be expected for relatively volatile analytes using the Turbovap.

### Method validation

The method validation showed that most of the analyzed compounds had a reasonable recovery in at least one of the methods (Table 2). Some compounds, such as anilines, yielded bad recovery percentages using both PLE and SPLE. These compounds are hard to analyze using GC in general. A method for LC-MS analysis is currently being developed that will be suitable for these compounds, as discussed above.

High recoveries (>100%) were observed for several compounds, e.g., low molecular weight PAHs and PCBs. For these compounds, the amount used to spike the samples before the extraction was rather low compared to typical concentrations found in sewage sludge. The natural occurrence of the analytes in the sludge, thus, could contribute to the amount detected. The fact that PCB 118 and PCB 105 yield high recovery percentages supports this hypothesis since they are major congeners in technical mixtures. Additionally, the recoveries of the 13C–labeled PCBs that were added as internal standards were calculated. The tetra- to hepta-CBs had recoveries between 71 and 102% for PLE and 84 and 110% for SPLE, which is fit for purpose for non-target screening methods.

However, for some polar compounds, such as bisphenol A and some other phenolics, recoveries and standard deviations were higher than acceptable for at least one of the methods. These high recoveries and variabilities could result from a matrix enhancement effect. When matrix is present, as in the sample, these matrix compounds can bind to active sites in the GC inlet system, column, or ion source making them unavailable for analytes. During instrument calibration, no such matrix compounds are introduced and therefore analytes can bind to active sites. This effect is called matrix induced chromatographic response enhancement (MICRE) and has been described previously [33, 34]. MICRE can be avoided by adding so-called analyte protectants to the sample and/or standard directly before analysis [35–37]. These analyte protectants bind to the active surfaces in the GC system, mainly the inlet, thereby facilitating analyte transfer. Since the use of analyte protectants could improve the analysis of polar compounds, the authors recommend the use of those for future analyses. In addition, more internal standards could be added allowing a better matching of analytes and internal standards.

It should be emphasized, however, that in non-target screening, it will never be possible to match analytes and internal standards perfectly and that reference compounds are lacking generally. Thus, the analyses will be semi-quantitative at best, although the relative concentrations among similar samples can be determined much more accurately. A tiered approach is therefore recommended: first, prioritize between the tentatively identified compounds, then confirm the identity of top-priority compounds, and finally develop quantitative analytical methods for the selected contaminants.

### Sulfur removal

Sulfur removal with copper and with TBA sulfite reagent produced similar results for the tested analytes. However, the variability among recoveries for copper treatment is lower and both the median as well as 10-percentile are higher. The US EPA stated in their method 3660B that treatment with copper powder (fine granular) may affect certain pesticides. Some of the mentioned pesticides (heptachlor, malathion, ethion, and diazinon) were included in the current study but no major losses could be seen. The recoveries for these analytes ranged from 82 to 92%. The reason for this may be that we used a more coarse copper granulate than what is recommended in method 3660B. The main advantage of the copper treatment, however, is its ease of use and speed. It will therefore be used in future suspect and non-target screening studies.

### Application

Applying the non-target screening methods to a sewage sludge sample revealed that both methods are very good at detecting non-target compounds. Although more compounds were identified using the PLE method (see Table 3), the SPLE method gave additional information. This is to be expected as the two methods cover different parts of the chemical property (polarity and size) space, as shown in Fig. 4.

Figure 3 clearly shows that there is a difference in polarity among compounds detected by the PLE and SPLE method, respectively. The majority of classified compounds that were found using the SPLE method were relatively non-polar compounds, such as alkanes or alkenes, alkyl-benzenes, alkyl-PAHs, and PAHs. In contrast, more polar compounds, such as alkanals, phthalates, fatty acids, and methyl derivatives, and long-chain amides were found using the PLE method. This can easily be explained by the loss of polar compounds due to sorption to the silica gel used in the SPLE method.

Size-dependent differences in compounds detected by the two methods are clearly shown in Fig. 2. The GC × GC region between 25 and 40 min first dimension retention time and 2.5 and 3.0 s second dimension retention time, which corresponds to the unresolved complex mixture (UCM) of crude oil or weathered petroleum, differs greatly for the two methods. The SPLE sample is much richer in UCM than the PLE sample, most likely due to losses of big molecules in the GPC step of the PLE method. A similar observation was made for the last part of the analysis (40 min or later): 26% of the compounds detected using the SPLE method (mainly steroids) eluted in this chromatographic region whereas only 16% of the compounds using the PLE method elute in this region. Vitamins E α and E γ, vitamin E α acetate, and 2,4-bis(2-phenylpropan-2-yl)phenol were also among the big compounds that were lost in the PLE method but found using the SPLE method (Table 4).

The non-target screening of sludge using the SPLE and PLE methods revealed many compounds besides the classified or grouped chemicals. Tables 4 and 5 show the compounds that could be tentatively identified. These compounds were primarily of anthropogenic origin (organophosphates, PPCPs, synthetic antioxidants, UV screens and stabilizers, pesticides and other chlorinated compounds, and process chemicals), which may be of environmental relevance, while some were of biogenic origin (e.g., extractives and vitamins). The tables appear to indicate that the PLE method revealed many more unique compounds than the SPLE method. However, many of the compounds that were grouped (PAHs, alkyl-PAHs, N/S/O-PAHs, steroids, and flavor and fragrances) could be tentatively identified using both methods (ESM, Table S6), and some were only tentatively identified using the SPLE method (e.g., large alkyl-benzenes). Hence, combining both methods increases the amount of information made available. The range of compounds that can be captured using the two non-target screening methods (Tables 4 and 5) is quite wide, including small and large compounds, e.g., m-amino-phenylacetylene (117 g/mol) and vitamin E α acetate (473 g/mol), as well as non-polar and relatively polar compounds (e.g., PCBs and organophosphate esters and phenols).

The results also illustrate how the NIST library can be used to identify compounds effectively by using both the similarity and probability scores. As shown in Table 3, the majority of tentatively identified compounds (83% for PLE and 91% for SPLE) were identified using the NIST library similarity scores, with a cutoff at 75% (tier 2). By using such a cutoff, only a limited number of spectra have to be manually reviewed but at the risk of losing information for low level contaminants affected by instrument noise. Some of these contaminants, those with distinct spectra, may be found by probability sorting the data that have similarity scores <75% (tier 3). In this way, 5 and 11% of the tentatively identified compounds using PLE and SPLE, respectively, could be revealed.

However, the majority of existing organic chemicals do not have an MS spectrum in the NIST library. Therefore, additional techniques are needed to identify compounds of particular concern, such as halogenated compounds. An attempt was made to screen for chlorinated and brominated compounds (tiers 4 and 5), as these compounds are generally environmentally relevant (c.f. PCBs, PBDEs, dioxins). Eleven compounds were found using halogen-specific filters (tier 4) and six compounds were found using mass defect plots (tier 5); all of these were chlorinated biphenyls. Molecular formula information for the tier 4 compounds could be generated using information about accurate mass and isotopic patterns. In some cases, searches in databases such as Chemspider and SciFinder resulted in a plausible candidate structure, e.g., 9,10-di(chloromethyl)-9,10-dihydroanthracene. In other cases, it was possible to extract a spectrum manually that was missed in the original peak picking and find a plausible hit in NIST. This resulted in the discovery of triphenylchloromethane, 9,10-di(chloromethyl)-anthracene, and *p*-(6-chloro-4-phenyl-2-quinolyl)aniline. Finally, an attempt was made to interpret the remaining spectra manually, resulting in two additional tentative identifications: one DDT metabolite and one chlorinated PAH. The spectra of all unknown chlorinated compounds that could not be assigned a tentative molecular structure were analyzed using MetFrag. This resulted in the identification of five additional compounds: (i) one compound which is likely to be dichloroxylenol, a commercial disinfectant [38], (ii) 4-(3,4-dichlorophenyl)tetralone and one of its isomers that are potential impurities in the pharmaceutical Sertraline [39], and (iii) two compounds belonging to the classes of dichlorophenyl coumarins or dichloroflavones, which are highlighted in patents suggesting their use in tire rubber production [40, 41]. To our knowledge, none of these compounds were detected in environmental samples up to now. Spectra for these compounds and other compounds that are not listed in NIST are given in the ESM (Figs. S1–S7).

Some of the other compounds that were tentatively identified have, to our knowledge, not previously been reported in sludge or in environmental samples either, e.g., the chlorinated PAH derivatives and triphenylchloromethane. However, similar compounds have been reported. Chlorinated PAHs have been found in incinerator flue gas, car exhaust, and urban air [42, 43]. Triphenylchloromethane is quite reactive and is frequently used in organic synthesis. It may be degraded to triphenylmethane that has been found in sediment [44].

Overall, the two proposed methods (PLE with GPC and SPLE, followed by sulfur removal with copper) in combination with a soon-to-be developed LC-MS method will provide a comprehensive methodology for the screening of a large variety of compounds with different properties in sewage sludge. This should also be feasible for other similar environmental matrices such as soil and sediment. It should be realized that there is an overlap between the applicability domains of the three methods. Once the methodology is fully developed, this should be highly advantageous. It should, for instance, be possible to use EI spectra from GC-MS to verify the identity of compounds originally detected by LC-MS and vice versa: it should be possible to use LC-MS data to generate molecular ion information that is often missing in GC-MS spectra. This could lead to the discovery of many more new and emerging chemicals in samples of environmental relevance, which could be subject to targeted measurement campaigns, environmental risk assessments, and STP improvement initiatives.

## Electronic supplementary material

ESM 1 (PDF 720 kb)
