# Supplementary material for: Methodology for non-target screening of sewage sludge using comprehensive two-dimensional gas chromatography coupled to high-resolution mass spectrometry
Source: Anal Bioanal Chem. 2017 Jun 23;409(20):4867–83. doi: 10.1007/s00216-017-0429-0 (PMC5519657; doi:10.1007/s00216-017-0429-0)
Supplement: Supplementary file 1 — (PDF 720 kb) [file 216_2017_429_MOESM1_ESM.pdf]

Analytical and Bioanalytical Chemistry

Electronic Supplementary Material

Methodology for non-target screening of sewage sludge using comprehensive two-dimensional gas chromatography coupled to high-resolution mass spectrometry

Cathrin Veenaas, Peter Haglund

Table S1 *MegaMix standard compounds including CAS-no., molecular formula and weight*

Compound	CAS no.	Formula	Exact mass
1,2,4-Trichlorobenzene	120-82-1	C6H3Cl3	179.930
1,2-Dichlorobenzene	95-50-1	C6H4Cl2	145.969
1,2-Dinitrobenzene	528-29-0	C6H4N2O4	168.017
1,3-Dichlorobenzene	541-73-1	C6H4Cl2	145.969
1,3-Dinitrobenzene	99-65-0	C6H4N2O4	168.017
1,4-Dichlorobenzene	106-46-7	C6H4Cl2	145.969
1,4-Dinitrobenzene	100-25-4	C6H4N2O4	168.017
1-Methylnaphthalene	90-12-0	C11H10	142.078
2,2′-Oxybis(1-chloropropane)	108-60-1	C6H12Cl2O	170.027
2,3,4,6-Tetrachlorophenol	58-90-2	C6H2Cl4O	229.886
2,3,5,6-Tetrachlorophenol	935-95-5	C6H2Cl4O	229.886
2,4,5-Trichlorophenol	95-95-4	C6H3Cl3O	195.925
2,4,6-Trichlorophenol	88-06-2	C6H3Cl3O	195.925
2,4-Dichlorophenol	120-83-2	C6H4Cl2O	161.964
2,4-Dimethylphenol	105-67-9	C8H10O	122.073
2,4-Dinitrophenol	51-28-5	C6H4N2O5	184.012
2,4-Dinitrotoluene	121-14-2	C7H6N2O4	182.033
2,6-Dinitrotoluene	606-20-2	C7H6N2O4	182.033
2-Chloronaphthalene	91-58-7	C10H7Cl	162.024
2-Chlorophenol	95-57-8	C6H5ClO	128.003
2-Methylnaphthalene	91-57-6	C11H10	142.078
2-Methylphenol (o-cresol)	95-48-7	C7H8O	108.058
2-Nitroaniline	88-74-4	C6H6N2O2	138.043
2-Nitrophenol	88-75-5	C6H5NO3	139.027
3-Methylphenol (m-cresol)*	108-39-4	C7H8O	108.058
3-Nitroaniline	99-09-2	C6H6N2O2	138.043
Dinitro-o-cresol	534-52-1	C7H6N2O5	198.028
4-Bromophenyl phenyl ether	101-55-3	C12H9BrO	247.984
4-Chloro-3-methylphenol	59-50-7	C7H7ClO	142.019
4-Chloroaniline	106-47-8	C6H6ClN	127.019
4-Chlorophenyl phenyl ether	7005-72-3	C12H9ClO	204.034
4-Methylphenol (p-cresol)*	106-44-5	C7H8O	108.058
4-Nitroaniline	100-01-6	C6H6N2O2	138.043
4-Nitrophenol	100-02-7	C6H5NO3	139.027
Acenaphthene	83-32-9	C12H10	154.078
Acenaphthylene	208-96-8	C12H8	152.063
Aniline	62-53-3	C6H7N	93.058
Anthracene	120-12-7	C14H10	178.078
Azobenzene	103-33-3	C12H10N2	182.084
Benz(a)anthracene	56-55-3	C18H12	228.094
Benzo(a)pyrene	50-32-8	C20H12	252.094
Benzo(b)fluoranthene	205-99-2	C20H12	252.094
Benzo(ghi)perylene	191-24-2	C22H12	276.094
Benzo(k)fluoranthene	207-08-9	C20H12	252.094

Compound	CAS no.	Formula	Exact mass
Benzyl alcohol	100-51-6	C7H8O	108.058
Benzyl butyl phthalate	85-68-7	C19H20O4	312.136
Bis(2-chloroethoxy)methane	111-91-1	C5H10Cl2O2	172.006
Bis(2-chloroethyl)ether	111-44-4	C4H8Cl2O	141.995
Bis(2-ethylhexyl)adipate	103-23-1	C22H42O4	370.308
Bis(2-ethylhexyl)phthalate	117-81-7	C24H38O4	390.277
Carbazole	86-74-8	C12H9N	167.074
Chrysene	218-01-9	C18H12	228.094
Dibenz(a,h)anthracene	53-70-3	C22H14	278.110
Dibenzofuran	132-64-9	C12H8O	168.058
Diethylphthalate	84-66-2	C12H14O4	222.089
Dimethylphthalate	131-11-3	C10H10O4	194.058
Di-n-butyl phthalate	84-74-2	C16H22O4	278.152
Di-n-octyl phthalate	117-84-0	C24H38O4	390.277
Diphenylamine[2]	122-39-4	C12H11N	169.089
Fluoranthene	206-44-0	C16H10	202.078
Fluorene	86-73-7	C13H10	166.078
Hexachlorobenzene	118-74-1	C6Cl6	281.813
Hexachlorobutadiene	87-68-3	C4Cl6	257.813
Hexachlorocyclopentadiene	77-47-4	C5Cl6	269.813
Hexachloroethane	67-72-1	C2Cl6	233.813
Indeno(1,2,3-cd)pyrene	193-39-5	C22H12	276.094
Isophorone	78-59-1	C9H14O	138.104
Naphthalene	91-20-3	C10H8	128.063
Nitrobenzene	98-95-3	C6H5NO2	123.032
N-Nitrosodimethylamine	62-75-9	C2H6N2O	74.048
N-Nitroso-di-n-propylamine	621-64-7	C6H14N2O	130.111
Pentachlorophenol	87-86-5	C6HCl5O	263.847
Phenanthrene	85-01-8	C14H10	178.078
Phenol	108-95-2	C6H6O	94.042
Pyrene	129-00-0	C16H10	202.078
Pyridine	110-86-1	C5H5N	79.042

Table S2 *Compounds and spiking levels of the deuterated PAH mix*

Compound	Amount spiked per sample (ng)
d8-Naphthalene	589.12
d8-Acenaphthylene	518.56
d10-Acenaphthene	589.12
d10-Phenanthrene	589.12
d10-Pyrene	550.08
d10-Fluorene	535.52
d12-Chrysene	589.12
d12-Perylene	589.12
d14-Dibenz(a,h)anthracene	589.12

Table S3 *Native standards used for validation experiment including supplier information and concentrations and corresponding internal standard (IS) that was used for calculations (CIL: Cambridge Isotope Laboratories)*

**Compound**	**Supplier**	**Corresponding IS**	**Amount spiked per sample (ng)**
MegaMix (see table 1)	RESTEK	PAHs, chlorophenols, chlorobenzenes	1834.8
PBDE 28, 154	Wellington Laboratories	13C PBDE 28 and 154	113.6
PBDE 47, 99, 153	Wellington Laboratories	13C PBDE 47, 99 and 153	113.1
Musk Xylene	Promochem	Musk Xylene D15	11245.7
Musk Ketone	Sigma Aldrich	Musk Xylene D15	556.6
Galaxolide	A2S through qmx	Tonalide D3	11169.1
Tonalide	Promochem GmbH	Tonalide D3	5769.8
PCB 77, 81, 123, 105, 114, 118, 156, 157, 167, 169, 189	CIL/Promochem	13C PCB 123, 123, 123, 114, 114, 118, 156, 157, 167, 189, 189	44.8
p,p'-DDE	PolyScience Corp. (Niles, III.)	13C PCB 123	853.0
Chlorpyrifos	Dr. Ehrenstorfer GmbH	Dacthal D6	826.4
Chlorthal-dimethyl/Dacthal	Dr. Ehrenstorfer GmbH	Dacthal D6	833.0
Diazinon	Dr. Ehrenstorfer GmbH	Musk Xylene D15	854.7
Triethylhexylphosphate	Dr. Ehrenstorfer GmbH	Triphenylphosphate D15	4790.1
2-Ethylhexyldiphenyl phosphate	Dr. Ehrenstorfer GmbH	Triphenylphosphate D15	5032.4
Tris(2-chloroethyl)phosphate	Dr. Ehrenstorfer GmbH	Tris(2-chloroethyl)phosphate D12	5918.8
Triphenylphosphate	Dr. Ehrenstorfer GmbH	Triphenylphosphate D15	3673.4
Tributylphosphate	Dr. Ehrenstorfer GmbH	Tributylphosphate D20	2980.1
Tris(1,3-dichloropropyl) phosphate	Dr. Ehrenstorfer GmbH	Triphenylphosphate D15	3707.0
Tris(2-butoxy-ethyl) phosphate	Dr. Ehrenstorfer GmbH	Triphenylphosphate D15	4340.8
Bisphenol A	Sigma Aldrich	Bisphenol A D16	5588.1
Triclosan	Fluka Biochemika	Triclosan D3	16928.6
Octachlorostyrene	CIL	Octachlorostyrene 13C	1348.7
Carbamazepin	Sigma Aldrich	Carbamazepin D10	1683.2

Table S4 *Labeled standards used for validation experiment including supplier information and concentrations (CIL: Cambridge Isotope Laboratories, N/A: no supplier information available)*

**Compound**	**Supplier**	**Amount spiked per sample (ng)**
PBDE 28 13C	Wellington laboratories	71.0
PBDE 47 13C	Wellington laboratories	74.1
PBDE 99 13C	Wellington laboratories	74.2
PBDE 153 13C	Wellington laboratories	72.7
PBDE 154 13C	Wellington laboratories	70.6
Pentachlorophenol 13C	Wellington laboratories	488.4
4-Chlorophenol 13C	Wellington laboratories	488.4
2,4-Dichlorophenol 13C	Wellington laboratories	488.4
2,4,5-Trichlorophenol 13C	Wellington laboratories	488.4
2,3,4,5-Tetrachlorophenol 13C	Wellington laboratories	488.4
Anthracene D10	CIL	91.7

Compound	Supplier	Amount spiked per sample (ng)
Benzo(k)fluoranthene D12	CIL	91.7
Acenaphthene D10	CIL	91.7
Chrysene D12	CIL	91.7
Fluorene D10	CIL	91.7
Naphthalene D8	CIL	91.7
Pyrene D10	CIL	91.7
Benzo(g,h,i)perylene D12	CIL	91.5
Acenaphthylene D8	CIL	87.3
Trichlorobenzene 13C	CIL	34.7
Tetrachlorobenzene 13C	CIL	34.7
Pentachlorobenzene 13C	CIL	34.7
Hexachlorobenzene 13C	CIL	34.7
Bisphenol A D16	Sigma Aldrich	1282.0
13C PCB 114, 118, 123, 156, 157, 167, 189	N/A	22.5
Dacthal D6	C/D/N Isotopes	637.2
Musk Xylene D15	LGC (Dr.Ehrenstorfer GmbH)	450.6
Tonalide D3	LGC (Dr.Ehrenstorfer GmbH)	695.6
Octachlorostyrene 13C	CIL	595.2
Triclosan D3	CDN Isotopes	1280.1
Tributylphosphate D20	N/A	15958.6
Tris(2-chloroethyl)phosphate D12	N/A	67026.1
Triphenylphosphate D15	N/A	2106.5
Carbamazepin D10	CIL	1366.1
RS PCB 97 13C	N/A	34.0
RS PCB 188 13C	N/A	52.4

Table S5 *Recovery values obtained during the method validation for the PLE method (PLE followed by GPC) and the SPLE method (selective PLE using silica as sorbent). Limit of detection (LOD) and quantification (LOQ) for the method (where possible) or instrument are given as well as information about the calibration curve.*

Compound	Recovery PLE method (%) ± StDev	Recovery SPLE method (%) ± StDev	LOD (ng/g)[a]	LOQ (ng/g) [a]	Linear range (ng/mL)	R^2
PAHs						
Naphthalene	135 ± 74	123 ± 54	2.85	8.62	0.56-1747.44	0.997
Acenaphthylene	136 ± 24	146 ± 12	0.42	1.26	0.56-1747.44	0.997
Acenaphthene	109 ± 33	90 ± 29	0.66	1.99	2.8-349.49	1.000
Dibenzofuran	110 ± 26	128 ± 28	0.73	2.22	2.8-1747.44	0.996
Fluorene	109 ± 32	110 ± 35	0.77	2.33	2.8-349.49	1.000
Phenanthrene	78 ± 39	121 ± 24	0.14	0.43	0.56-349.49	1.000
Anthracene	110 ± 29	154 ± 7	0.1	0.32	2.8-349.49	1.000
Fluoranthene	95 ± 7	94 ± 32	0.3	0.92	2.8-349.49	1.000
Pyrene	70 ± 6	68 ± 79	PLE: 0.26<br>SPLE: 0.34	PLE: 0.78<br>SPLE: 1.04	2.8-1747.44	0.998
Benz(a)anthracene	123 ± 22	185 ± 6	2.76	8.38	13.98-1747.44	0.997
Chrysene	79 ± 3	143 ± 3	0.76	2.31	2.8-349.49	1.000
Benzo(b+k) fluoranthene	69 ± 3	104 ± 1	3.24	9.83	13.98-1747.44	1.000
Benzo(a)pyrene	< LOQ	82 ± 87	1.6	4.85	13.98-1747.44	0.999

Compound	Recovery PLE method (%) ± StDev	Recovery SPLE method (%) ± StDev	LOD (ng/g)[a]	LOQ (ng/g)[a]	Linear range (ng/mL)	R^2
Indeno(1,2,3-c,d)pyrene	< LOQ	68 ± 16	9.42	28.55	21.55-107.74	1.000
Dibenz (a,h) anthracene	< LOQ	73 ± 8	7.56	22.91	13.98-1747.44	0.999
Benzo (g,h,i) perylene	< LOQ	80 ± 16	76.45	231.67	69.9-1747.44	1.000
PCBs						
PCB 81	64 ± 12	50 ± 24	0.45	1.36	8.54-42.68	1.000
PCB 77	112 ± 28	143 ± 30	0.62	1.89	One-point calibration	
PCB 123	108 ± 4	120 ± 26	0.33	0.99	8.54-42.68	1.000
PCB 118	149 ± 6	125 ± 10	0.34	1.04	8.54-42.68	1.000
PCB 114	130 ± 4	107 ± 24	1.09	3.31	One-point calibration	
PCB 105	186 ± 12	95 ± 23	0.13	0.4	8.54-42.68	1.000
PCB 167	219 ± 2	106 ± 13	0.13	0.39	8.54-42.68	1.000
PCB 156	173 ± 4	127 ± 23	0.24	0.73	8.54-42.68	1.000
PCB 157	105 ± 3	141 ± 11	0.35	1.05	8.54-42.68	1.000
PCB 189	123 ± 9	122 ± 16	0.22	0.66	One-point calibration	
Diphenyl ethers						
4-Chlorodiphenyl ether	106 ± 36	106 ± 24	0.51	1.54	13.98-349.49	1.000
4-Bromodiphenyl ether	98 ± 11	99 ± 26	0.07	0.2	13.98-1747.44	1.000
BDE-28	99 ± 3	126 ± 15	0.59	1.79	21.64-108.18	1.000
BDE-47	107 ± 3	129 ± 62	4.52	13.69	21.55-107.74	1.000
BDE-99	87 ± 10	110 ± 8	2.16	6.54	21.55-107.74	1.000
BDE-154	132 ± 21	123 ± 1	24.41	73.98	One-point calibration	
BDE-153	122 ± 59	< LOQ	8.68	26.3	One-point calibration	
Phenolics						
2,4-Dimethylphenol	211 ± 138	56 ± 43	1.47	4.45	2.8-1747.44	0.999
2,4-Dichlorophenol	177 ± 126	37 ± 40	1.56	4.74	13.98-1747.44	0.999
Bisphenol A	198 ± 100	< LOQ	129.43	392.2	8.52-5322.03	0.999
4-Chloro-3-methylphenol	111 ± 24	< LOQ	2.29	6.95	13.98-1747.44	1.000
2,4,6-Trichlorophenol	48 ± 2	287 ± 136	2.02	6.13	13.98-1747.44	1.000
2,4,5-Trichlorophenol	110 ± 3	186 ± 172	3.27	9.91	13.98-1747.44	1.000
2,3,5,6-Tetrachlorophenol	36 ± 5	< LOQ	7.95	24.09	13.98-1747.44	1.000
2,3,4,6-Tetrachlorophenol	49 ± 3	< LOQ	12.37	37.5	13.98-1747.44	0.999
Pentachlorophenol	101 ± 10	< LOQ	9.5	28.79	69.9-1747.44	1.000
Other non-polar compounds						
Trichlorobenzene	135 ± 64	111 ± 53	0.65	1.96	2.8-1747.44	0.998
Hexachlorobenzene	111 ± 3	108 ± 2	1.36	4.12	2.8-1747.44	1.000
Bis(2-chloroethoxy)methane	91 ± 71	61 ± 55	0.02	0.05	2.8-349.49	0.997
Hexachloro-1,3-butadiene	127 ± 54	110 ± 39	0.75	2.29	13.98-1747.44	0.995
Octachlorostyrene	114 ± 2	111 ± 6	0.2	0.62	10.28-1284.49	0.999
N-Compounds						
2,6-Dinitrotoluene	134 ± 15	101 ± 9	1.82	5.5	69.9-1747.44	1.000
1,2-Dinitrobenzene	28 ± 2	11 ± 2	3.57	10.82	69.9-1747.44	0.999
Azobenzene	133 ± 38	137 ± 45	2.74	8.3	13.98-1747.44	0.999
Diphenylamine	117 ± 34	135 ± 51	0.04	0.12	2.8-1747.44	0.999
Phthalates						
Dimethylphthalate	101 ± 16	< LOQ	0.33	1.01	2.8-1747.44	0.997
Diethyl phthalate	88 ± 31	2 ± 1	19.14	58	0.56-349.49	1.000
Dibutyl phthalate	43 ± 15	4 ± 12	PLE: 49.6<br>SPLE: 75.99	PLE: 150.5<br>SPLE: 230.26	0.56-349.49	0.999
Benzyl butyl phthalate	85 ± 6	< LOQ	10.04	30.42	13.98-1747.44	1.000
Phosphate						
Tributylphosphate	99 ± 19	< LOQ	83.07	251.74	22.71-2838.2	0.996
TCEP[b]	21 ± 2	< LOQ	26.2	79.41	45.1-1127.4	1.000
TDCPP[b]	57 ± 2	< LOQ	52.71	159.72	141.22-706.1	1.000
Triphenylphosphate	59 ± 2	< LOQ	1.58	4.8	27.99-699.7	0.999
TBEP[b]	59 ± 13	< LOQ	8.11	24.57	165.36-4134.12	0.999

Compound	Recovery PLE method (%) ± StDev	Recovery SPLE method (%) ± StDev	LOD (ng/g)[a]	LOQ (ng/g) [a]	Linear range (ng/mL)	R^2
EHDPP[b]	51 ± 11	< LOQ	PLE: 0.36<br>SPLE: 0.23	PLE: 1.09<br>SPLE: 0.68	38.34-4792.75	0.999
Triethylhexylphosphate	3 ± 5	< LOQ	10.01	30.33	7.3-4561.98	0.998
Pesticides, pharma						
Diazinon	73 ± 2	< LOQ	12.4	37.57	6.51-162.8	1.000
Chlorpyrifos	81 ± 9	43 ± 15	6.64	20.11	31.48-787.07	1.000
Dacthal	92 ± 3	96 ± 7	3.11	9.41	31.73-793.33	1.000
p,p'-DDE	72 ± 7	133 ± 23	0.11	0.34	6.5-162.48	1.000
Triclosan	78 ± 12	104 ± 5	6.22	18.86	25.8-3224.5	1.000
Carbamazepine	95 ± 3	< LOQ	11.07	33.55	64.12-1603.03	1.000
Fragrances						
Isophorone	103 ± 78	< LOQ	2.01	6.09	13.98-1747.44	1.000
Galaxolide	30 ± 28	108 ± 61	0.83	2.53	17.02-2127.45	0.999
Tonalide	90 ± 28	145 ± 19	28.04	84.97	8.79-5495.07	0.996
Musk Xylene	65 ± 8	96 ± 36	7.64	23.14	85.68-2142.04	0.999
Musk Ketone	137 ± 22	49 ± 30	7.21	21.85	106.02-530.12	1.000

[a] For compounds with different LOD or LOQ the values were calculated from the blank values. For compounds where only one value is given the value was determined using the standard dilution curve and is therefore an instrument LOD or LOQ, respectively, only.

[b] Abbreviations: TCEP: Tris(2-chloroethyl)phosphate, TDCPP: Tris(1,3-dichloropropyl) phosphate, TBEP: Tris(2-butoxy-ethyl) phosphate, EHDPP: 2-Ethylhexyldiphenyl phosphate.

Table S6 *Tentatively identified compounds (Tiers 2 and 3) detected in the final extracts from PLE or SPLE methods that were included among the grouped compounds (**Figure 3**). The compounds are sorted by retention time.*

Name	PLE	SPLE	RT 1 (s)	RT 2 (s)	MW (amu)	Mass dev. (ppm)	Rev. Sim.	Proba-bility	CAS no.	IUPAC name
Anethole	x		626	3.35	148	0.19	918	6144	104-46-1	1-Methoxy-4-(1-propen-1-yl)benzene
Carvacrol	x		630	3.23	150	0.76	772	2171	499-75-2	5-Isopropyl-2-methylphenol
2-Methylnaphthalene		x	642	2.93	142	-0.49	771	1259	91-57-6	2-Methylnaphthalene
Indole	x	x	642	3.88	117	0.58	953	6222	120-72-9	1H-Indole
3-Isopropyl-5-methylphenol	x		646	3.26	150	0.84	821	2340	3228-03-3	3-Isopropyl-5-methylphenol
1-Methylnaphthalene	x		670	3.55	142	0.35	881	3576	90-12-0	1-Methylnaphthalene
Heptylcyclohexane		x	710	2.78	182	0.33	823	683	5617-41-4	Heptylcyclohexane
4-Phenyl-3-buten-2-one	x		738	3.62	146	-3.09	688	7178	122-57-6	4-Phenyl-3-buten-2-one
Biphenyl	x		774	3.55	154	-0.22	886	2611	92-52-4	Biphenyl
3-Methyl-1H-indole	x	x	786	3.85	131	-0.25	931	2938	83-34-1	3-Methyl-1H-indole
1,2,3,4-Tetrahydro-5,6-dimethylnaphthalene	x		794	3.29	160	-1.17	809	2129	20027-77-4	5,6-Dimethyl-1,2,3,4-tetrahydronaphthalene
2-Ethylnaphthalene	x		798	3.45	156	0.34	833	4733	939-27-5	2-Ethylnaphthalene
1-Ethylnaphthalene	x		802	3.53	156	1.38	758	2488	1127-76-0	1-Ethylnaphthalene
Diphenyl ether	x		810	3.53	170	-0.15	868	6848	101-84-8	1,1'-Oxydibenzene
2,6-Dimethylnaphthalene	x	x	814	3.45	156	-0.14	921	1102	581-42-0	2,6-Dimethylnaphthalene
1-Methyl-2-cyclohexylbenzene	x		834	3.21	174	-2.20	795	7010	4501-35-3	1-Cyclohexyl-2-methylbenzene
Verdyl acetate	x	x	838	3.30	192	2.72	751	9704	5413-60-5	Tricyclo[5.2.1.02,6]dec-3-en-8-yl acetate
1,3-Dimethylnaphthalene	x		838	3.50	156	-0.38	903	1094	575-41-7	1,3-Dimethylnaphthalene
cis-Cinnamic acid	x		838	3.55	148	-1.08	766	4754	102-94-3	(2Z)-3-Phenylacrylic acid
Caryophyllene	x		842	2.98	204	0.57	880	1750	87-44-5	(1R,9S)-4,11,11-Trimethyl-8-methylenebicyclo[7.2.0]undec-4-ene
1,2,3,4-Tetrahydro-1,5,7-trimethylnaphthalene	x		842	3.18	174	-0.47	699	224	21693-55-0	1,5,7-Trimethyl-1,2,3,4-tetrahydronaphthalene
1,7-Dimethylnaphthalene	x		842	3.51	156	-0.18	889	1848	575-37-1	1,7-Dimethylnaphthalene
α-Ionone	x		846	3.14	192	1.46	789	5663	127-41-3	(3E)-4-(2,6,6-Trimethyl-2-cyclohexen-1-yl)-3-buten-2-one
1,2,3,4-Tetrahydro-1,5,8-trimethylnaphthalene	x		850	3.22	174	-1.93	708	1164	21693-51-6	1,5,8-Trimethyl-1,2,3,4-tetrahydronaphthalene
Cinnamic acid		x	854	3.48	148	-0.44	904	3585	621-82-9	(2E)-3-Phenylacrylic acid
Diphenylmethane	x		854	3.54	168	-1.23	818	1663	101-81-5	1,1'-Methylenedibenzene
4-(2,6,6-Trimethyl-1-cyclohexen-1-yl)-2-butanone	x		862	3.13	194	N/A	752	7141	17283-81-7	4-(2,6,6-Trimethyl-1-cyclohexen-1-yl)-2-butanone
2,3-Dimethylnaphthalene	x		866	3.54	156	-0.28	861	1710	581-40-8	2,3-Dimethylnaphthalene
1,2,3,4-Tetrahydro-1,6,8-trimethylnaphthalene	x		870	3.23	174	-0.96	734	418	30316-36-0	1,6,8-Trimethyl-1,2,3,4-tetrahydronaphthalene
1,2,3,4-Tetrahydro-2,5,8-trimethylnaphthalene	x		878	3.24	174	-1.05	738	2093	30316-17-7	2,5,8-Trimethyl-1,2,3,4-tetrahydronaphthalene
1,2-Dimethylnaphthalene	x	x	890	3.58	156	-1.69	843	1392	573-98-8	1,2-Dimethylnaphthalene
Oxindole	x		918	4.22	133	0.2	886	1344	59-48-3	1,3-Dihydro-2H-indol-2-one

Name	PLE	SPLE	RT 1 (s)	RT 2 (s)	MW (amu)	Mass dev. (ppm)	Rev. Sim.	Proba-bility	CAS no.	IUPAC name
Curcumene	x	x	926	3.03	202	1.61	858	7212	644-30-4	1-Methyl-4-(6-methyl-5-hepten-2-yl)benzene
ß-Ionone	x		934	3.20	192	0.15	899	4650	14901-07-6	(3E)-4-(2,6,6-Trimethyl-1-cyclohexen-1-yl)-3-buten-2-one
4-Methylbiphenyl	x	x	934	3.49	168	-0.63	820	2801	644-08-6	4-Methylbiphenyl
Acenaphthene	x	x	934	3.75	154	-1.18	885	7481	83-32-9	1,2-Dihydroacenaphthylene
1-Propylnaphthalene	x		938	3.42	170	0.02	822	6880	2765-18-6	1-Propylnaphthalene
2-Ethylbiphenyl	x		946	3.40	182	0.22	761	212	1812-51-7	2-Ethylbiphenyl
3-Methylbiphenyl	x	x	946	3.52	168	-4.97	828	996	643-93-6	3-Methylbiphenyl
α-Muurolene	x		958	3.01	204	0.64	761	509	31983-22-9	(1S,4aS,8aR)-1-Isopropyl-4,7-dimethyl-1,2,4a,5,6,8a-hexahydronaphthalene
C3-Naphthalene	x	x	962	3.42	170	0.23	811	1425	-	-
ß-Bisabolene	x		966	2.94	204	0.67	889	2887	495-61-4	1-Methyl-4-(6-methyl-2-heptanyl)cyclohexane
[1-(2,4-cyclopentadien-1-ylidene)ethyl]benzene	x		974	3.62	168	2.03	737	8334	2320-32-3	[1-(2,4-Cyclopentadien-1-ylidene)ethyl]benzene
C3-Naphthalene	x		978	3.48	170	0.39	747	805	-	-
Hexahydro-4,7-methano-1H-indenol	x	x	982	3.30	150	0.89	757	78	37275-49-3	Tricyclo[5.2.1.02,6]dec-1-en-3-ol
Dibenzofuran	x	x	982	3.70	168	0.47	907	8868	132-64-9	Dibenzo[b,d]furan
1-Naphthalenol	x		986	3.89	144	-0.14	865	2136	90-15-3	1-Naphthol
δ-Cadinene	x		990	3.02	204	0.87	826	466	483-76-1	(1S,8aR)-1-Isopropyl-4,7-dimethyl-1,2,3,5,6,8a-hexahydronaphthalene
Calamenene I	x	x	990	3.14	202	0.29	820	2550	483-77-2	4-Isopropyl-1,6-dimethyl-1,2,3,4-tetrahydronaphthalene
2-Ethoxynaphthalene	x	x	990	3.59	172	0.66	881	5054	93-18-5	2-Ethoxynaphthalene
Lilial	x	x	994	3.30	204	-0.18	804	6989	80-54-6	2-Methyl-3-[4-(2-methyl-2-propanyl)phenyl]propanal
C3-Naphthalene	x	x	994	3.45	170	0.21	859	1160	-	-
Isoamyl salicylate	x		1002	3.21	208	1.02	869	1482	87-20-7	3-Methylbutyl salicylate
Dihydroactinidiolide	x		1002	3.84	180	1.13	886	5540	17092-92-1	4,4,7a-Trimethyl-5,6,7,7a-tetrahydro-1-benzofuran-2(4H)-one
C3-Naphthalene	x	x	1026	3.48	170	-0.21	890	3465	-	-
2,2,2-Trichloro-1-phenylethyl acetate	x	x	1038	3.45	266	N/A	712	7836	90-17-5	2,2,2-Trichloro-1-phenylethyl acetate
C3-Naphthalene		x	1050	3.49	170	-0.25	860	3056	-	-
2-Methylbutyl salicylate	x		1058	3.23	208	0.16	876	3711	51115-63-0	2-Methylbutyl salicylate
C3-Naphthalene	x	x	1074	3.57	170	1.09	769	3176	-	-
Fluorene	x	x	1074	3.78	166	-1.18	917	6559	86-73-7	9H-Fluorene
3-Tetradecanone	x		1078	2.92	212	N/A	732	7151	629-23-2	3-Tetradecanone
Caryophyllene oxide	x		1078	3.24	220	N/A	843	4511	1139-30-6	(1R,4R,6R,10S)-4,12,12-Trimethyl-9-methylene-5-oxatricyclo[8.2.0.04,6]dodecane
C2-Biphenyl	x	x	1082	3.46	182	-0.25	835	1671	-	-
C3-Naphthalene	x		1082	3.60	170	1.13	838	5698	-	-
C2-Biphenyl	x	x	1098	3.48	182	-0.22	842	1978	-	-
1,4-Dihydrofluorene	x	x	1102	3.72	168	-1.11	787	636	41593-21-9	4,9-Dihydro-1H-fluorene
2-(Methylthio)benzothiazole	x		1106	4.00	181	1.17	756	9133	615-22-5	2-(Methylsulfanyl)-1,3-benzothiazole
9-Methylfluorene	x		1106	3.67	180	0.14	769	1643	2523-37-7	9-Methyl-9H-fluorene

Name	PLE	SPLE	RT 1 (s)	RT 2 (s)	MW (amu)	Mass dev. (ppm)	Rev. Sim.	Proba-bility	CAS no.	IUPAC name
Acetylnaphthalene	x		1118	3.91	170	-0.22	775	3936	941-98-0	1-(1-Naphthyl)ethanone
2-Methyl-1-naphthalenol	x		1122	3.94	158	0.19	860	7473	7469-77-4	2-Methyl-1-naphthol
C4-Naphthalene	x		1126	3.43	184	1.17	739	1600	-	-
C2-Biphenyl	x		1134	3.54	182	0.35	777	2990	-	-
(1-Butylheptyl)benzene		x	1138	2.87	232	0.91	902	8975	4537-15-9	5-Undecanylbenzene
4-Methyldibenzofuran	x	x	1146	3.65	182	0.02	824	307	7320-53-8	4-Methyldibenzo[b,d]furan
Diphenylmethanol	x		1146	3.88	184	-1.59	862	8231	91-01-0	Diphenylmethanol
Vanillyl acetone	x		1158	3.79	194	1.33	857	6562	303187-89-5	4-(4-Hydroxy-3-methoxyphenyl)-2-butanone
9H-Xanthene	x	x	1162	3.70	182	0.51	810	4545	92-83-1	9H-Xanthene
Methyl dihydrojasmonate	x		1166	3.31	226	N/A	723	7123	24851-98-7	Methyl 2-(3-oxo-2-pentylcyclopentyl)acetate
C4-Naphthalene	x	x	1166	3.44	184	1.17	795	993	-	-
(1-Ethylnonyl)benzene		x	1178	2.91	232	0.42	809	7857	4536-87-2	3-Undecanylbenzene
C4-Naphthalene	x	x	1194	3.46	184	0.29	826	4620	-	-
Diisopropylnaphthalene	x		1198	3.27	212	-0.19	811	2061	-	-
γ-Undecalactone	x		1198	3.38	184	N/A	822	2172	104-67-6	5-Heptyloxolan-2-one
Diisopropylnaphthalene	x	x	1206	3.28	212	0.17	894	7527	-	-
C4-Biphenyl	x	x	1206	3.44	210	0.82	803	6087	-	-
(1-Methyldecyl)benzene	x	x	1230	2.95	232	1.26	738	3731	4536-88-3	2-Undecanylbenzene
2-Methyl-9H-fluorene	x	x	1230	3.71	180	0.22	895	2026	1430-97-3	2-Methyl-9H-fluorene
C4-Biphenyl	x	x	1242	3.49	210	1.32	765	5875	-	-
3-Methyl-9H-fluorene	x	x	1242	3.74	180	-0.03	762	1643	2523-39-9	3-Methyl-9H-fluorene
3-Hydroxybiphenyl	x		1250	3.92	170	0.09	755	418	580-51-8	3-Biphenylol
Diisopropylnaphthalene	x	x	1254	3.28	212	0.13	828	6614	-	-
C2-Biphenyl	x	x	1254	3.65	182	0.13	780	3269	-	-
1,1,3-trimethyl-3-phenyl-indan	x	x	1258	3.38	236	1.12	830	5127	3910-35-8	1,1,3-Trimethyl-3-phenylindane
(1-Pentylheptyl)benzene	x	x	1262	2.90	246	0.31	724	7129	2719-62-2	6-Dodecanylbenzene
C2-Biphenyl	x	x	1262	3.67	182	-1.17	702	2333	-	-
Diisopropylnaphthalene	x	x	1266	3.26	212	-0.14	896	7458	-	-
(1-Butyloctyl)benzene	x	x	1270	2.91	246	0.70	641	3572	2719-63-3	5-Dodecanylbenzene
Diisopropylnaphthalene	x	x	1270	3.41	212	0.30	806	6476	-	-
C2-Biphenyl	x	x	1270	3.80	182	-1.17	726	1609	-	-
1-Benzyl-3,5-dimethylbenzene	x	x	1274	3.53	196	1.12	733	2732	28122-27-2	1-Benzyl-3,5-dimethylbenzene
C4-Biphenyl	x	x	1298	3.52	210	0.49	884	6368	-	-
Naphtho[2,1-b]thiophene	x	x	1306	4.03	184	-0.08	911	4323	233-02-3	Naphtho[2,1-b]thiophene
Benzyl Benzoate	x	x	1314	3.76	212	0.71	841	8196	120-51-4	Benzyl Benzoate
Phenanthrene	x	x	1342	4.03	178	0.38	937	3509	85-01-8	Phenanthrene
Anthracene	x	x	1354	4.02	178	0.38	903	1873	120-12-7	Anthracene
(1-Methylundecyl)benzene	x	x	1358	2.95	246	2.76	793	3383	2719-61-1	2-Dodecanylbenzene
C2-9H-Fluorene	x	x	1378	3.67	194	0.52	881	3468	-	-
(1-Pentyloctyl)benzene		x	1386	2.87	260	1.48	866	5078	4534-49-0	6-Tridecanylbenzene

Name	PLE	SPLE	RT 1 (s)	RT 2 (s)	MW (amu)	Mass dev. (ppm)	Rev. Sim.	Proba-bility	CAS no.	IUPAC name
C2-9H-Fluorene	x		1386	3.69	194	0.72	835	3481	-	-
5,7-Dihydrodibenz[c,e]oxepin	x		1386	3.93	196	1.02	791	791	1136-22-7	5,7-Dihydrodibenzo[c,e]oxepine
(1-Butylnonyl)benzene		x	1394	2.87	260	1.7	894	9073	4534-50-3	5-Tridecanylbenzene
C2-9H-Fluorene		x	1398	3.67	194	-1.17	891	5802	-	-
(1-Propyldecyl)benzene		x	1410	2.88	260	1.25	859	8656	4534-51-4	4-Tridecanylbenzene
Carbazole	x	x	1414	4.29	167	-0.28	833	821	86-74-8	9H-Carbazole
C2-9H-Fluorene	x	x	1414	3.76	194	0.33	858	5594	-	-
Galaxolide	x	x	1426	3.33	258	0.49	911	8862	1222-05-5	4,6,6,7,8,8-Hexamethyl-1,3,4,6,7,8-hexahydrocyclopenta[g]isochromene
4-Methylnaphtho[1,2-b]thiophene	x	x	1426	3.96	198	-0.37	836	3309	67388-11-8	4-Methylnaphtho[1,2-b]thiophene
1-(Phenylmethylene)-1H-indene	x	x	1434	3.88	204	-0.17	917	3842	5394-86-5	1-Benzylidene-1H-indene
(1-Ethylundecyl)benzene	x	x	1438	2.93	260	1.50	724	4885	4534-52-5	3-Tridecanylbenzene
Tonalid	x	x	1442	3.26	258	0.69	900	4259	21145-77-7	1-(3,5,5,6,8,8-hexamethyl-5,6,7,8-tetrahydro-2-naphthalenyl)ethanone
Benzyl salicylate	x		1446	3.72	228	0.22	866	5336	118-58-1	Benzyl salicylate
4-Methyldibenzothiophene	x	x	1454	3.96	198	-1.04	774	1052	7372-88-5	4-Methyldibenzothiophene
Galaxolide impurity (1)[a]	x	x	1458	3.38	(258)	(1.00)	(854)	(8728)	-	-
Galaxolide impurity (2)[a]	x	x	1466	3.39	(258)	(0.22)	(845)	(8736)	-	-
Methylphenanthrene/anthracene	x	x	1478	3.93	192	-0.27	888	1291	-	-
Versalide	x	x	1482	3.45	258	0.54	773	475	88-29-9	1-(3-Ethyl-5,5,8,8-tetramethyl-5,6,7,8-tetrahydro-2-naphthalenyl)ethanone
3-Methyldibenzothiophene	x	x	1486	4.04	198	-0.29	772	2951	16587-52-3	3-Methyldibenzothiophene
(1-Methyldodecyl)benzene	x	x	1486	2.91	260	0.93	768	3483	4534-53-6	2-Tridecanylbenzene
Methylphenanthrene/anthracene		x	1486	3.93	192	-0.58	913	1714	-	-
Methylphenanthrene/anthracene	x	x	1494	3.95	192	-0.62	900	1656	-	-
Galaxolide impurity (3)[a]	x	x	1498	3.44	(258)	(0.65)	(876)	(8976)	-	-
2-Methyl-9H-carbazole		x	1506	4.11	181	-0.67	784	2147	3652-91-3	2-Methyl-9H-carbazole
Methylphenanthrene/anthracene	x		1510	4.03	192	-0.44	793	1342	-	-
Phenanthrindene	x	x	1510	4.11	190	-0.67	868	6057	203-64-5	4H-Cyclopenta[def]phenanthrene
Methylphenanthrene/anthracene	x		1518	4.03	192	0.55	900	1542	-	-
4-Methylcarbazole	x	x	1546	4.21	181	-0.31	950	3169	3770-48-7	4-Methyl-9H-carbazole
4,6-Dimethyldibenzothiophene	x	x	1546	3.90	212	0.26	859	980	1207-12-1	4,6-Dimethyldibenzo[b,d]thiophene
2-Phenylnaphthalene	x	x	1566	3.97	204	0.78	883	1601	612-94-2	2-Phenylnaphthalene
2,6-Dimethyldibenzothiophene	x	x	1574	3.90	212	-0.2	827	678	89816-75-1	2,6-Dimethyldibenzo[b,d]thiophene
C2-phenanthrene/anthracene	x	x	1586	3.86	206	2.45	757	4028	-	-
2,8-Dimethyldibenzothiophene	x	x	1594	3.95	212	-0.41	559	122	1207-15-4	2,8-Dimethyldibenzo[b,d]thiophene
3,7-Dimethyldibenzothiophene	x	x	1602	3.97	212	-0.06	919	3210	1136-85-2	3,7-Dimethyldibenzo[b,d]thiophene
C2-phenanthrene/anthracene	x	x	1606	3.88	206	0.02	873	3264	-	-
C2-phenanthrene/anthracene	x	x	1618	3.87	206	0.13	859	2465	-	-
2,7-Dimethyldibenzothiophene	x	x	1618	3.98	212	-0.14	736	1833	31317-19-8	2,7-Dimethyldibenzo[b,d]thiophene

Name	PLE	SPLE	RT 1 (s)	RT 2 (s)	MW (amu)	Mass dev. (ppm)	Rev. Sim.	Proba-bility	CAS no.	IUPAC name
18-norabieta-8,11,13-triene	x	x	1622	3.42	256	0.16	813	6837	5323-56-8	7-Isopropyl-1,4a-dimethyl-1,2,3,4,4a,9,10,10a-octahydrophenanthrene
C2-phenanthrene/anthracene	x		1630	3.91	206	0.49	803	1769	-	-
p-Dicyclohexylbenzene	x		1638	3.41	242	-0.25	732	7619	1087-02-1	1,4-Dicyclohexylbenzene
C2-phenanthrene/anthracene	x	x	1638	3.93	206	0.39	918	3234	-	-
10,18-Bisnorabieta-8,11,13-triene		x	1650	3.39	242	0.58	798	7162	32624-67-2	7-Isopropyl-1-methyl-1,2,3,4,4a,9,10,10a-octahydrophenanthrene
2,2,5,5-Tetrachloro-3-(dichloromethyl)-4-(dichloromethylene)cyclopentanol		x	1650	3.46	384	3.76	0	0	1027939-45-2	2,2,5,5-Tetrachloro-3-(dichloromethyl)-4-(dichloromethylene)cyclopentanol
C2-phenanthrene/anthracene	x	x	1654	3.97	206	0.39	857	2178	-	-
Dihydropyrene	x	x	1662	4.08	204	-1.66	842	1534	-	-
C2-phenanthrene/anthracene		x	1662	3.92	206	0.03	826	2121	-	-
Dihydropyrene		x	1666	4.06	204	0.05	669	379	-	-
C2-phenanthrene/anthracene	x	x	1666	4.00	206	0.01	769	2837	-	-
Fluoranthene	x	x	1674	4.23	202	0.73	933	5121	206-44-0	Fluoranthene
2,3-Dihydro-1H-cyclopenta[l]phenanthrene	x	x	1690	3.89	218	-1.28	790	1752	723-98-8	2,3-Dihydro-1H-cyclopenta[l]phenanthrene
C2-phenanthrene/anthracene	x		1702	4.04	206	-0.03	758	2414	-	-
Dibenzopentalene	x	x	1702	4.29	202	1.51	712	5	248-58-8	Indeno[2,1-a]indene
Dihydro-Benz[de]anthracene	x		1702	3.91	218	-0.53	635	4450	-	-
Phenaleno[1,9-bc]thiophene	x	x	1714	4.41	208	0.3	867	9252	79965-99-4	Phenaleno[1,9-bc]thiophene
3-Phenyl-2,3-dihydro-1H-indene-1,2-dione	x	x	1722	4.27	222	-1.05	780	4225	92438-99-8	3-Phenyl-1H-indene-1,2(3H)-dione
1-Ethyl-2-methylphenanthrene		x	1722	3.77	220	0	757	7232	61983-53-7	1-Ethyl-2-methylphenanthrene
Pyrene	x	x	1730	4.42	202	0.27	894	3690	129-00-0	Pyrene
Benzo[b]naphtho[2,3-d]furan	x	x	1738	4.13	218	0.95	822	234	243-42-5	Benzo[b]naphtho[2,3-d]furan
Dihydropyrene	x	x	1738	4.27	204	-0.92	914	2086	-	-
C3-Phenanthrene	x	x	1738	3.86	220	1.24	788	5160	-	-
Benzo[b]naphtho[1,2-d]furan	x	x	1758	4.20	218	0.26	877	3628	205-39-0	Benzo[b]naphtho[1,2-d]furan
C3-Phenanthrene	x	x	1758	3.86	220	0.75	811	8059	-	-
C3-Phenanthrene		x	1770	3.83	220	0.05	878	7991	-	-
1-(Phenylmethoxy)naphthalene	x	x	1774	4.05	234	-0.37	879	8928	607-58-9	1-(Benzyloxy)naphthalene
Benzo[kl]xanthene	x	x	1774	4.19	218	-0.07	876	4031	200-23-7	Benzo[kl]xanthene
C3-Phenanthrene		x	1782	3.86	220	0.54	822	7832	-	-
C3-Phenanthrene	x		1790	3.91	220	0.04	805	8104	-	-
C3-Phenanthrene	x		1798	3.94	220	-0.97	791	7128	-	-
11H-Benzo[a]fluorene		x	1810	4.11	216	-0.51	696	210	238-84-6	11H-Benzo[a]fluorene
C3-Phenanthrene	x		1810	3.97	220	0.07	774	7138	-	-
4H-Benzo[def]carbazole	x		1814	4.77	191	-0.46	839	7151	203-65-6	4H-Benzo[def]carbazole
7H-Benzo[c]fluorene	x	x	1834	4.28	216	-2.95	926	3227	205-12-9	7H-Benzo[c]fluorene
2-Isopropyl-10-methylphenanthrene	x	x	1834	3.82	234	0.3	859	5008	66552-97-4	2-Isopropyl-10-methylphenanthrene

Name	PLE	SPLE	RT 1 (s)	RT 2 (s)	MW (amu)	Mass dev. (ppm)	Rev. Sim.	Proba-bility	CAS no.	IUPAC name
Androst-16-en-3-ol	x		1838	3.66	274	0.59	819	6581	-	-
Androst-16-en-3-ol	x		1846	3.70	274	0.62	869	6981	-	-
C4-Dibenzo[b,d]thiophene	x		1846	3.82	240	0.20	638	7071	-	-
7H-Benzanthrene	x	x	1854	4.28	216	-0.98	893	3780	199-94-0	7H-Benzo[de]anthracene
Methylpyrene	x		1862	4.32	216	-0.41	924	5425	-	-
2H-Phenanthro[9,10-b]pyran	x	x	1870	4.08	232	-0.28	882	9555	217-67-4	2H-Dibenzo[f,h]chromene
Dihydro-Benz[de]anthracene	x		1870	4.33	218	0.7	854	4580	-	-
Dihydro-Benz[de]anthracene	x		1882	4.24	218	-0.73	807	3103	-	-
1H-Phenanthro[9,10-c]pyran	x		1886	4.14	232	-0.8	868	9841	217-62-9	-
Methylpyrene	x	x	1886	4.40	216	0.12	935	5602	-	-
Methylpyrene	x	x	1894	4.40	216	-0.41	912	4905	-	-
C4-Dibenzo[b,d]thiophene	x		1898	3.95	240	-0.53	738	7311	-	-
4-(2-Phenylethenyl)benzenamine	x		1914	4.17	265	N/A	780	3174	85650-52-8	4-[(E)-2-Phenylvinyl]aniline
γ-Octadecalactone	x		1922	3.30	282	N/A	846	3829	502-26-1	5-Tetradecyldihydro-2(3H)-furanone
1-Cyclopentyl-4-(3-cyclopentylpropyl)dodecane		x	1930	2.78	348	-1.57	704	342	7225-68-5	[4-(3-Cyclopentylpropyl)dodecyl]cyclopentane
Dehydroisoandrosterone acetate	x		1930	3.96	330	N/A	846	3558	853-23-6	17-Oxoandrost-5-en-3-yl acetate
8-Isopropyl-1,3-dimethylphenanthrene	x		1938	3.75	248	0.31	781	9320	135886-06-5	8-Isopropyl-1,3-dimethylphenanthrene
Dihydrochrysene	x	x	1958	4.21	230	0.4	837	5571	-	-
11H-Benzo[a]fluoren-11-one	x		1978	4.54	230	-0.32	809	1615	479-79-8	11H-Benzo[a]fluoren-11-one
C2-Pyrene	x	x	1982	4.26	230	0.07	861	4889	-	-
Dihydrochrysene	x	x	1998	4.29	230	0.65	776	1853	-	-
C2-Pyrene	x	x	2006	4.32	230	-0.28	813	2878	-	-
Benzo[b]naphtho[2,1-d]thiophene	x	x	2006	4.52	234	0.08	886	2474	239-35-0	Benzo[b]naphtho[2,1-d]thiophene
Benzo[ghi]fluoranthene	x	x	2018	4.56	226	0.57	820	4259	203-12-3	Benzo[ghi]fluoranthene
Bis(1-phenylethyl)phenol	x		2026	4.07	302	0.10	705	7200	-	-
Benz[c]acridine	x		2026	4.58	229	-0.23	820	7505	225-51-4	Benzo[c]acridine
C2-Pyrene	x		2030	4.39	230	0.20	830	4298	-	-
Phenanthro[4,3-b]thiophene	x		2030	4.59	234	0.78	757	1261	195-68-6	Phenanthro[4,3-b]thiophene
Benzo[b]naphtho[1,2-d]thiophene	x	x	2050	4.58	234	0.52	848	3246	205-43-6	Benzo[b]naphtho[1,2-d]thiophene
Benz[a]anthracene	x	x	2070	4.58	228	0.41	940	5875	56-55-3	Tetraphene
Chrysene	x	x	2082	4.65	228	0.22	851	98	218-01-9	Chrysene
Benzo(c)carbazole	x		2086	4.77	217	0.71	919	3756	34777-33-8	7H-Benzo[c]carbazole
3,4-Dihydrocyclopenta[cd]pyrene	x	x	2102	4.70	228	-1.13	882	7140	25732-74-5	3,4-Dihydrocyclopenta[cd]pyrene
Methylbenzo[b]naphtho[2,1-d]thiophene	x	x	2106	4.45	248	-0.39	852	4586	-	1-Methylbenzo[b]naphtho[2,1-d]thiophene
Androsterone	x		2118	4.16	290	0.02	806	3598	53-42-9	(3α,5α)-3-Hydroxyandrostan-17-one
Methylbenzo[b]naphtho[2,1-d]thiophene	x	x	2126	4.47	248	0.1	846	866	-	-
Naphtho[2,1,8,7-klmn]xanthene	x		2134	4.66	242	0.7	868	9359	191-37-7	Naphtho[2,1,8,7-klmn]xanthene
11H-Benzo[a]carbazole	x		2134	4.98	217	1.11	881	3271	239-01-0	11H-Benzo[a]carbazole
7,12-Dihydro-2-methylbenz[a]anthracene	x		2138	4.29	244	0.04	692	7002	35187-44-1	2-Methyl-7,12-dihydrotetraphene

Name	PLE	SPLE	RT 1 (s)	RT 2 (s)	MW (amu)	Mass dev. (ppm)	Rev. Sim.	Proba-bility	CAS no.	IUPAC name
Methylbenzo[b]naphtho[2,1-d]thiophene	x	x	2154	4.55	248	-0.46	846	3288	-	-
Epiandrosterone	x		2158	4.27	290	0.38	903	6400	481-29-8	(3β,5α)-3-Hydroxyandrostan-17-one
Methyl-Benz[a]anthracene/chrysene	x	x	2190	4.53	242	0.34	895	1493	-	-
Methyl-Benz[a]anthracene/chrysene	x	x	2202	4.60	242	0.06	863	908	-	-
Cyclopenta[a]pyrene	x		2210	4.78	240	-3.89	841	8761	-	-
C2-Benzo[b]naphtho[2,3-d]thiophene	x	x	2214	4.40	262	0.90	757	7767	-	-
Methyl-Benz[a]anthracene/chrysene	x		2214	4.60	242	-1.42	815	1737	-	-
9,10-Dihydro-9,10-dimethylbenz[a]anthracene	x		2222	4.19	258	1.11	691	7047	-	9,10-Dimethyl-9,10-dihydrobenz[a]anthracene
C2-Benzo[b]naphtho[2,3-d]thiophene	x	x	2222	4.44	262	0.52	722	6696	-	-
Methyl-Benz[a]anthracene/chrysene	x		2226	4.73	242	-0.69	808	1836	-	-
Cyclopenta[a]pyrene		x	2230	4.74	240	-2.03	835	9148	-	-
C2-Benzo[b]naphtho[2,3-d]thiophene	x	x	2242	4.44	262	0.38	645	7609	-	-
C2-Benzo[b]naphtho[2,3-d]thiophene	x	x	2250	4.51	262	0.33	741	7982	-	-
2-Phenylphenanthrene	x		2254	4.61	254	0.96	785	2067	4325-77-3	2-Phenylphenanthrene
C2-Benzo[b]naphtho[2,3-d]thiophene	x	x	2270	4.53	262	1.15	790	8260	-	-
Pregnanolone	x		2282	4.12	318	-0.06	846	4072	128-20-1	(3α,5β)-3-Hydroxypregnan-20-one
C2-Benz(a)anthracene/Chrysene		x	2298	4.41	256	0.65	767	2348	-	-
C2-Benz(a)anthracene/Chrysene	x		2306	4.54	256	0.54	775	2024	-	-
4,12-Dimethylbenz[a]anthracene	x		2314	4.59	256	0.68	802	1526	35187-19-0	4,12-Dimethyltetraphene
Allopregnanolone	x		2318	4.22	318	0.85	855	4162	516-55-2	(3α,5α)-3-Hydroxypregnan-20-one
5α-Dihydroprogesterone	x		2354	4.38	316	0.78	868	5974	566-65-4	(5α)-Pregnane-3,20-dione
Benz[b]fluoranthene	x		2362	4.95	252	0.93	858	879	205-99-2	Benzo[e]acephenanthrylene
(5β)-Cholest-3-ene		x	2370	3.28	370	2.39	759	1284	13901-20-7	(5R,8S,9S,10S,13R,14S,17R)-10,13-dimethyl-17-[(2R)-6-methylheptan-2-yl]-2,5,6,7,8,9,11,12,14,15,16,17-dodecahydro-1H-cyclopenta[a]phenanthrene
Benzo[e]pyrene		x	2426	5.17	252	0.18	929	2518	192-97-2	Benzo[e]pyrene
Benzo[a]pyrene		x	2434	5.22	252	0.82	916	1088	50-32-8	Benzo[pqr]tetraphene
(3β)-Cholesta-4,6-dien-3-ol		x	2438	3.46	384	N/A	712	8068	14214-69-8	(8S,9S,10R,13R,14S,17R)-10,13-dimethyl-17-[(2R)-6-methylheptan-2-yl]-2,3,8,9,11,12,14,15,16,17-decahydro-1H-cyclopenta[a]phenanthren-3-ol
Perylene		x	2458	5.34	252	1.91	889	1657	198-55-0	Perylene
13H-Dibenzo[a,h]fluorene		x	2470	4.88	266	-0.54	831	3511	239-85-0	13H-Dibenzo[a,h]fluorene
A-Neooleana-3(5),12-diene	x	x	2474	3.55	408	2.56	798	8110	22586-84-1	3-Isopropyl-5a,5b,7a,10,10,13b-hexamethyl-2,4,5,5a,5b,6,7,7a,8,9,10,11,11a,13,13a,13b-hexadecahydro-1H-cyclopenta[a]chrysene
5α-Ergost-8(14)-ene	x		2502	3.57	384	0.54	792	4141	6673-69-4	(5R,9R,10S,13R,17R)-17-[(2R,5S)-5,6-dimethylheptan-2-yl]-10,13-dimethyl-2,3,4,5,6,7,9,11,12,15,16,17-dodecahydro-1H-cyclopenta[a]phenanthrene
10-Methylbenzo(a)pyrene		x	2526	5.55	266	-0.13	821	5836	63104-32-5	10-Methylbenzo[pqr]tetraphene

Name	PLE	SPLE	RT 1 (s)	RT 2 (s)	MW (amu)	Mass dev. (ppm)	Rev. Sim.	Proba-bility	CAS no.	IUPAC name
11H-Indeno[2,1-a]phenanthrene		x	2554	5.79	266	1.35	759	3533	220-97-3	11H-Indeno[2,1-a]phenanthrene
Ergostanol	x		2570	4.56	402	0.07	635	7823	-	-
Ergostanol	x		2582	4.56	402	1.70	559	7235	-	-
(5β,22E)-Ergost-22-en-3-one	x	x	2670	4.57	398	0.55	747	7905	18865-44-6	(17R)-17-[(E,2R,5R)-5,6-dimethylhept-3-en-2-yl]-10,13-dimethyl-1,2,4,5,6,7,8,9,11,12,14,15,16,17-tetradecahydrocyclopenta[a]phenanthren-3-one
Ergostanol	x	x	2682	4.55	402	2.07	846	7995	-	-
Ergostanol		x	2690	4.54	402	1.27	799	7990	-	-
4α-Methylcholest-7-en-3-one	x		2710	4.85	398	0.01	661	9168	13490-57-8	(4S,9R,10S,13R,14R,17R)-4,10,13-trimethyl-17-[(2R)-6-methylheptan-2-yl]-1,2,4,5,6,9,11,12,14,15,16,17-dodecahydrocyclopenta[a]phenanthren-3-one
Stigmasta-5,24(28)-dien-3-ol	x		2714	5.02	412	N/A	681	7347	-	-
Ergostanol		x	2722	4.76	402	-4.13	627	8148	-	-
Ergostanol	x		2726	4.89	402	-0.65	753	9553	-	-
Cholest-4-en-3-one	x		2742	5.59	384	1.58	625	8300	601-57-0	Cholest-4-en-3-one
Stigmastanol	x	x	2758	4.93	416	1.93	737	7862	19466-47-8	(3β)-Stigmastan-3-ol
24-Methyldesmosterol	x		2770	5.45	398	-0.88	611	7689	20780-41-0	(3β)-Ergosta-5,24-dien-3-ol
(3β)-Lanost-8-en-3-ol		x	2778	5.04	428	0.52	618	8030	79-62-9	(3β)-Lanost-8-en-3-ol
Lanosta-8,24-dien-3-one	x	x	2790	5.45	424	0.34	696	7144	5539-04-8	(5ξ)-Lanosta-8,24-dien-3-one
Stigmasta-5,24(28)-dien-3-ol	x		2806	5.50	412	2.54	713	7127	-	-
Cycloartanol		x	2830	5.47	428	6.3	527	7060	4657-58-3	9,19-Cyclolanostan-3-ol
ß-Amyrin	x	x	2838	6.16	426	N/A	554	210	559-70-6	(3β)-Olean-12-en-3-ol
28-Norolean-17-en-3-one	x	x	2842	6.50	410	1.25	872	8798	5912-72-1	(4aR,6aR,6aR,6bR,14aR,14bR)-4,4,6a,6b,11,11,14b-heptamethyl-1,2,4a,5,6,6a,7,8,9,10,12,13,14,14a-tetradecahydropicen-3-one
(3β)-9,19-Cyclolanost-24-en-3-ol		x	2874	6.02	426	-0.13	848	6668	469-38-5	(3β,9β)-9,19-Cyclolanost-24-en-3-ol
α-Amyrin	x	x	2886	6.64	426	1.22	720	4107	638-95-9	(3β)-Urs-12-en-3-ol
Cholest-4-ene-3,6-dione	x		2890	6.77	398	1.83	733	9338	984-84-9	Cholest-4-ene-3,6-dione
Arundoin		x	2902	6.14	440	0.62	852	8601	4555-56-0	(3R,3aR,5aR,5bR,7aR,9S,11aS,13aS,13bR)-3-Isopropyl-9-methoxy-3a,5a,8,8,11a,13a-hexamethyl-2,3,3a,4,5,5a,5b,6,7,7a,8,9,10,11,11a,13,13a,13b-octadecahydro-1H-cyclopenta[a]chrysene
7-Oxocholesterol	x		2918	7.30	400	1.03	706	7775	566-28-9	3-Hydroxycholest-5-en-7-one
24-Methylenecycloartan-3-one	x	x	2922	6.30	438	1.34	737	4265	1449-08-7	24-Methylene-9,19-cyclolanostan-3-one

[a] These compounds show a spectrum very similar to Galaxolide (see **Figures S8 – S10**). A patent issued in 1987 [1] mentions three known impurities of Galaxolide. Those impurity compounds have the same molecular weight as Galaxolide and a similar structure. The spectra are, therefore, assumed to look similar to that of Galaxolide.

Table S7 *Tentatively identified compounds (Tiers 2 and 3) detected in the final extracts from the PLE or SPLE methods, but not included among the classified or grouped compounds (****Figure 3****).*

Name	RT 1 (s)	RT 2 (s)	MW (amu)	CAS no.	IUPAC name
Alkyl-phenols					
2,3,6-Trimethylphenol	598	3.35	136	2416-94-6	2,3,6-Trimethylphenol
4-*tert*-Octylphenol	1102	3.26	206	140-66-9	4-(2,4,4-Trimethyl-2-pentanyl)phenol
4-(1-Phenylethyl)phenol	1258	3.78	198	1988-89-2	4-(1-Phenylethyl)phenol
4-(1,1-Dimethylhexyl)phenol	1274	3.26	206	30784-29-3	4-(2-Methyl-2-heptanyl)phenol
3-(2-Phenylethyl)phenol	1394	3.85	198	33675-75-1	3-(2-Phenylethyl)phenol
Extractives					
10,18-Bisnorabieta-5,7,9(10),11,13-pentaene	1706	3.58	238	6566-19-4	7-Isopropyl-1-methyl-1,2,3,4-tetrahydrophenanthrene
Dehydroabietal	1890	3.73	284	13601-88-2	Abieta-8,11,13-trien-18-al
Ferruginol	1946	3.67	286	514-62-5	Abieta-8,11,13-trien-12-ol
Methyl dehydroabietate	1958	3.62	314	1235-74-1	Methyl abieta-8,11,13-trien-18-oate
4-Epidehydroabietol	1986	3.82	286	24035-43-6	[(1S,4aS,10aR)-1,4a-dimethyl-7-propan-2-yl-2,3,4,9,10,10a-hexahydrophenanthren-1-yl]methanol
Dehydroabietic acid	2054	3.83	300	1740-19-8	Abieta-8(14),9(11),12-trien-18-oic acid
Organophosphate esters					
Tris(1,3-dichloroisopropyl)phosphate (TCPP)	1950	3.74	428	13674-87-8	Tris(1,3-dichloro-2-propanyl) phosphate
Triphenyl phosphate (TPP)	2018	4.21	326	115-86-6	Triphenyl phosphate
2-Ethylhexyl diphenyl phosphate (EHDPP)	2038	3.61	362	1241-94-7	2-Ethylhexyl diphenyl phosphate
Cresyl diphenyl phosphate (CDPP, 2 isomers)	2098	4.17	340	5254-12-6	4-Methylphenyl diphenyl phosphate
Isopropyl-phenyl diphenyl phosphate (iPrDPP)	2142	3.99	368	-	2-Isopropylphenyl diphenyl phosphate
Dicresyl phenyl phosphate (DCPP, 2 isomers)	2174	4.13	354	-	-
Tricresyl phosphate (TCP, 3 isomers)	2246	4.16	368	-	-
PPCP					
2-(Dodecyloxy)ethanol	1246	2.96	230	4536-30-5	2-(Dodecyloxy)ethanol
Diphenylmethoxy acetic acid	1422	4.11	242	21409-25-6	(Diphenylmethoxy)acetic acid
Clorophene	1482	3.90	218	120-32-1	2-Benzyl-4-chlorophenol
1-Dodecyl-2-pyrrolidinone	1726	3.34	253	2687-96-9	1-Dodecyl-2-pyrrolidinone
Bromhexidine	2030	3.85	374	3572-43-8	2,4-Dibromo-6-{[cyclohexyl(methyl)amino]methyl}aniline
Phenyl tetradecyl carbonate	2090	3.28	334	959283-58-0	Phenyl tetradecyl carbonate
2-Palmitoylglycerol	2106	3.28	330	23470-00-0	1,3-Dihydroxy-2-propanyl palmitate
Dronabinol	2126	3.71	314	1972-08-3	(6aR,10aR)-6,6,9-Trimethyl-3-pentyl-6a,7,8,10a-tetrahydro-6H-benzo[c]chromen-1-ol
Cannabinol	2190	3.78	310	521-35-7	6,6,9-Trimethyl-3-pentyl-6H-benzo[c]chromen-1-ol
Clozapine	2494	5.08	326	5786-21-0	8-Chloro-11-(4-methyl-1-piperazinyl)-5H-dibenzo[b,e][1,4]diazepine
Stabilizers, antioxidants					
1-(4-*tert*-Butylphenyl)propan-2-one	878	3.37	190	81561-77-5	1-[4-(2-Methyl-2-propanyl)phenyl]acetone
Butylated hydroxytoluene (BHT)	970	3.09	220	128-37-0	4-Methyl-2,6-bis(2-methyl-2-propanyl)phenol
tert-Octyldiphenylamine	1894	3.60	281	27177-37-3	N-Phenyl-4-(2,4,4-trimethyl-2-pentanyl)aniline
4,4'-Di-*tert*-butyl-diphenylamine	1914	3.53	281	4627-22-9	4-(2-Methyl-2-propanyl)-N-[4-(2-methyl-2-propanyl)phenyl]aniline
2,6-Bis(1-phenylethyl)phenol	2042	4.11	302	4237-28-9	2,6-Bis(1-phenylethyl)phenol
2,4-Bis(1-phenylethyl)phenol	2098	4.10	302	2769-94-0	2,4-Bis(1-phenylethyl)phenol

Name	RT 1 (s)	RT 2 (s)	MW (amu)	CAS no.	IUPAC name
N,N'-Diphenyl-1,4-benzenediamine	2270	4.78	260	74-31-7	N,N'-Diphenyl-1,4-benzenediamine
4-Octyl-N-(4-octylphenyl)benzenamine	2486	3.73	393	101-67-7	4-Octyl-N-(4-octylphenyl)aniline
Vitamin E γ	2570	3.77	416	7616-22-0	2,7,8-Trimethyl-2-(4,8,12-trimethyltridecyl)-6-chromanol
Vitamin E α	2630	3.97	430	59-02-9	(2R)-2,5,7,8-Tetramethyl-2-[(4R,8R)-4,8,12-trimethyltridecyl]-6-chromanol
Vitamin E α acetate	2678	4.02	472	7695-91-2	[2,5,7,8-tetramethyl-2-(4,8,12-trimethyltridecyl)-3,4-dihydrochromen-6-yl] acetate
Stabilizers/screens, UV					
Benzophenone	1138	3.85	182	119-61-9	Diphenylmethanone
2-Ethylhexyl salicylate	1362	3.14	250	118-60-5	2-Ethylhexyl salicylate
Phenyl cinnamonitrile	1454	4.06	205	3531-24-6	3,3-Diphenylacrylonitrile
Homosalate	1462	3.23	262	118-56-9	3,3,5-Trimethylcyclohexyl salicylate
Oxybenzone	1630	3.99	228	131-57-7	(2-Hydroxy-4-methoxyphenyl)(phenyl)methanone
Tinuvin P	1670	3.90	225	2440-22-4	2-(2H-Benzotriazol-2-yl)-4-methylphenol
2-Ethylhexyl *trans*-4-methoxycinnamate	1930	3.44	290	83834-59-7	2-Ethylhexyl (2E)-3-(4-methoxyphenyl)acrylate
Tinuvin 326	2154	3.70	315	3896-11-5	2-(5-Chloro-2H-benzotriazol-2-yl)-4-methyl-6-(2-methyl-2-propanyl)phenol
Octocrylene	2258	3.76	361	6197-30-4	2-Ethylhexyl 2-cyano-3,3-diphenylacrylate
Other halogenated compounds					
2,3-Dichlorobenzenamine	702	3.71	161	608-27-5	2,3-Dichloroaniline
4-Chloro-m-xylenol	782	3.46	156	88-04-0	4-Chlor-3,5-dimethylphenol
2,3,4-Trichlorobenzenamine	1014	3.81	195	634-67-3	2,3,4-Trichloroaniline
4-Iodophenylacetonitrile	1298	4.45	243	51628-12-7	(4-Iodophenyl)acetonitrile
p,*p*'-DDD	1814	3.87	318	72-54-8	1,1'-(2,2-Dichloro-1,1-ethanediyl)bis(4-chlorobenzene)
6,7-Dichloro-4b,10-ethenobenz(a)azulene	1898	0.01	272	110190-25-5	12,13-Dichlorotetracyclo[6.6.2.$0^{1,9}$.$0^{2,7}$]hexadeca-2,4,6,9,11,13,15-heptaene
trans-Permethrin	2282	3.89	390	61949-77-7	3-Phenoxybenzyl (1R,3S)-3-(2,2-dichlorovinyl)-2,2-dimethylcyclopropanecarboxylate
Other process chemicals					
m-Aminophenylacetylene	638	3.38	117	54060-30-9	3-Ethynylaniline
2,3,6,7-Tetramethylquinoxaline	1194	3.68	186	6957-19-3	2,3,6,7-Tetramethylquinoxaline
2,4-Diphenyl-4-methyl-1-pentene	1346	3.46	236	6362-80-7	1,1'-(4-Methyl-1-pentene-2,4-diyl)dibenzene
2,4-Diphenyl-4-methyl-2(*E*)-pentene	1398	3.43	236	22768-22-5	1,1'-[(2E)-4-Methyl-2-pentene-2,4-diyl]dibenzene
4-Methoxydibenzyl	1438	3.78	212	14310-21-5	1-Methoxy-4-(2-phenylethyl)benzene
Hexadecanenitrile	1470	3.04	237	629-79-8	Hexadecanenitrile
Diphenyl sulfone	1518	4.43	218	127-63-9	1,1'-Sulfonyldibenzene
2-Mercaptobenzothiazole	1558	4.79	167	149-30-4	1,3-Benzothiazole-2(3H)-thione
4-Stilbenol	1646	4.01	196	6554-98-9	4-[(E)-2-Phenylvinyl]phenol
Isopropylthioxanthone (ITX)	1998	4.22	254	5495-84-1	2-Isopropyl-9H-thioxanthen-9-one
4-Benzoylbiphenyl	2070	4.31	258	2128-93-0	4-Biphenylyl(phenyl)methanone
2,4-Bis(2-phenylpropan-2-yl)phenol	2114	3.92	330	2772-45-4	2,4-Bis(2-phenyl-2-propanyl)phenol
Tier 4 and 5 compounds					
Dichloroxylenol	798	3.42	190	88-04-0	4-Chlor-3,5-dimethylphenol
PCB 92	1682	3.72	324	52663-61-3	2,2',3,5,5'-Pentachlorobiphenyl
DDMS (DDT metabolite)	1702	3.79	284	2642-80-0	1,1'-(2-Chloro-1,1-ethanediyl)bis(4-chlorobenzene)
PCB 101	1730	3.63	324	37680-73-2	2,2',4,5,5'-Pentachlorobiphenyl

**Name**	**RT 1 (s)**	**RT 2 (s)**	**MW (amu)**	**CAS no.**	**IUPAC name**
Triphenylchloromethane	1762	3.93	278	76-83-5	1,1',1"-(Chloromethanetriyl)tribenzene1,1',1"-(Chloromethanetriyl)tribenzene
9,10-Di(chloromethyl)-9,10- dihydroanthracene	1806	3.85	276	18777-37-2	9,10-Bis(chloromethyl)-9,10-dihydroanthracene
PCB 151	1838	3.69	358	52663-63-5	2,2',3,5,5',6-Hexachlorobiphenyl
PCB 149	1866	3.74	358	38380-04-0	2,2',3,4',5',6-Hexachlorobiphenyl
PCB 153	1918	3.52	358	35694-04-3	2,2',4,4',5,5'-Hexachlorobiphenyl
PCB 138	1978	3.83	358	35065-28-2	2,2',3,4,4',5'-Hexachlorobiphenyl
4-(3,4-Dichlorophenyl)tetralone	2042	4.33	290	79560-19-3	4-(3,4-dichlorophenyl)-3,4-dihydro-1(2H)-naphthalenone
9,10-Di(chloromethyl)anthracene	2374	4.84	274	10387-13-0	9,10-Bis(chloromethyl)anthracene
p-(6-Chloro-4-phenyl-2-quinolyl)aniline	2578	5.73	330	21923-52-4	4-(6-Chloro-4-phenyl-2-quinolinyl)aniline

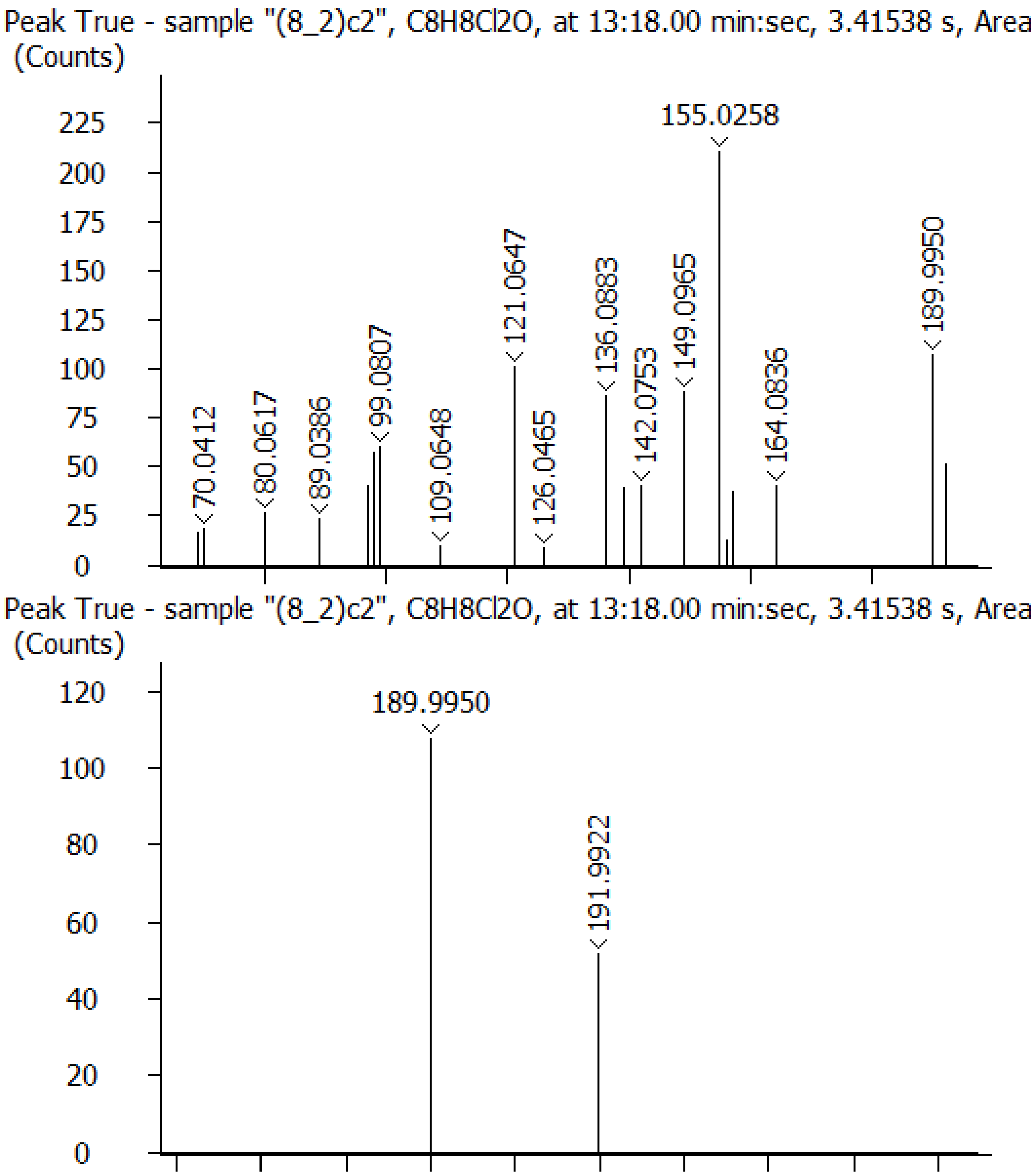

Figure S1 *Peak "Dichloroxylenol", RT 1 (s): 798, RT 2 (s): 3.42; top: whole spectrum, bottom: highest mass fragment*

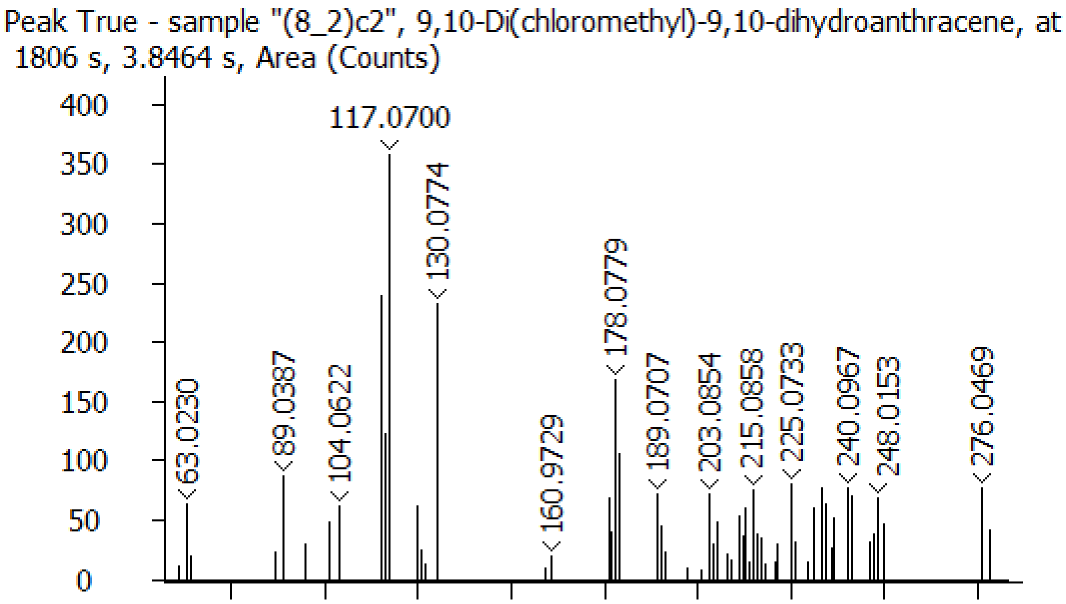

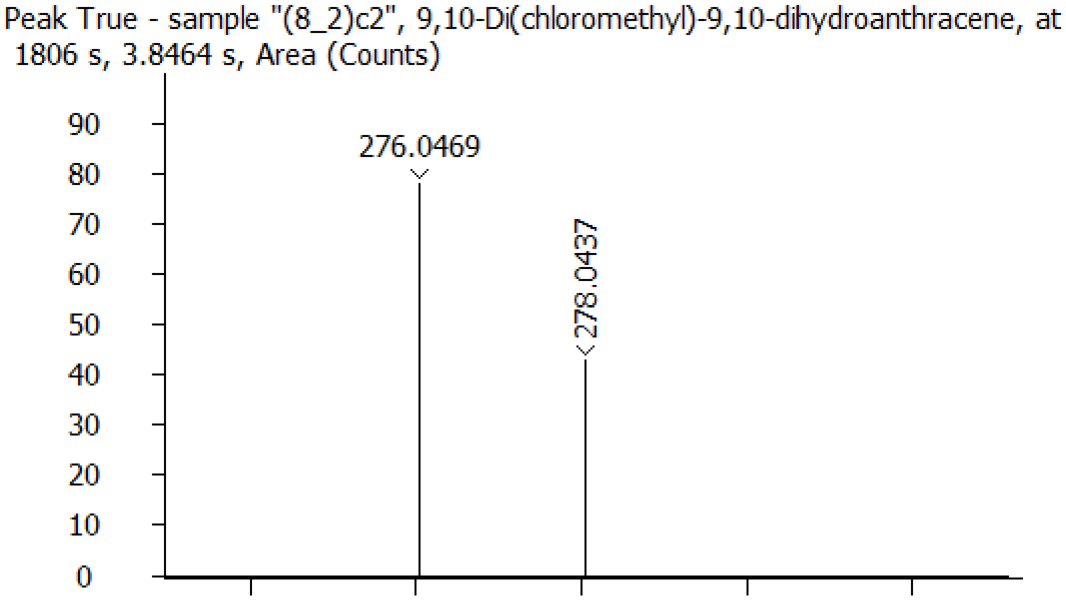

Figure S2 *Peak "9,10-Di(chloromethyl)-9,10- dihydroanthracene ", RT 1 (s): 1806, RT 2 (s): 3.85; top: whole spectrum, bottom: highest mass fragment*

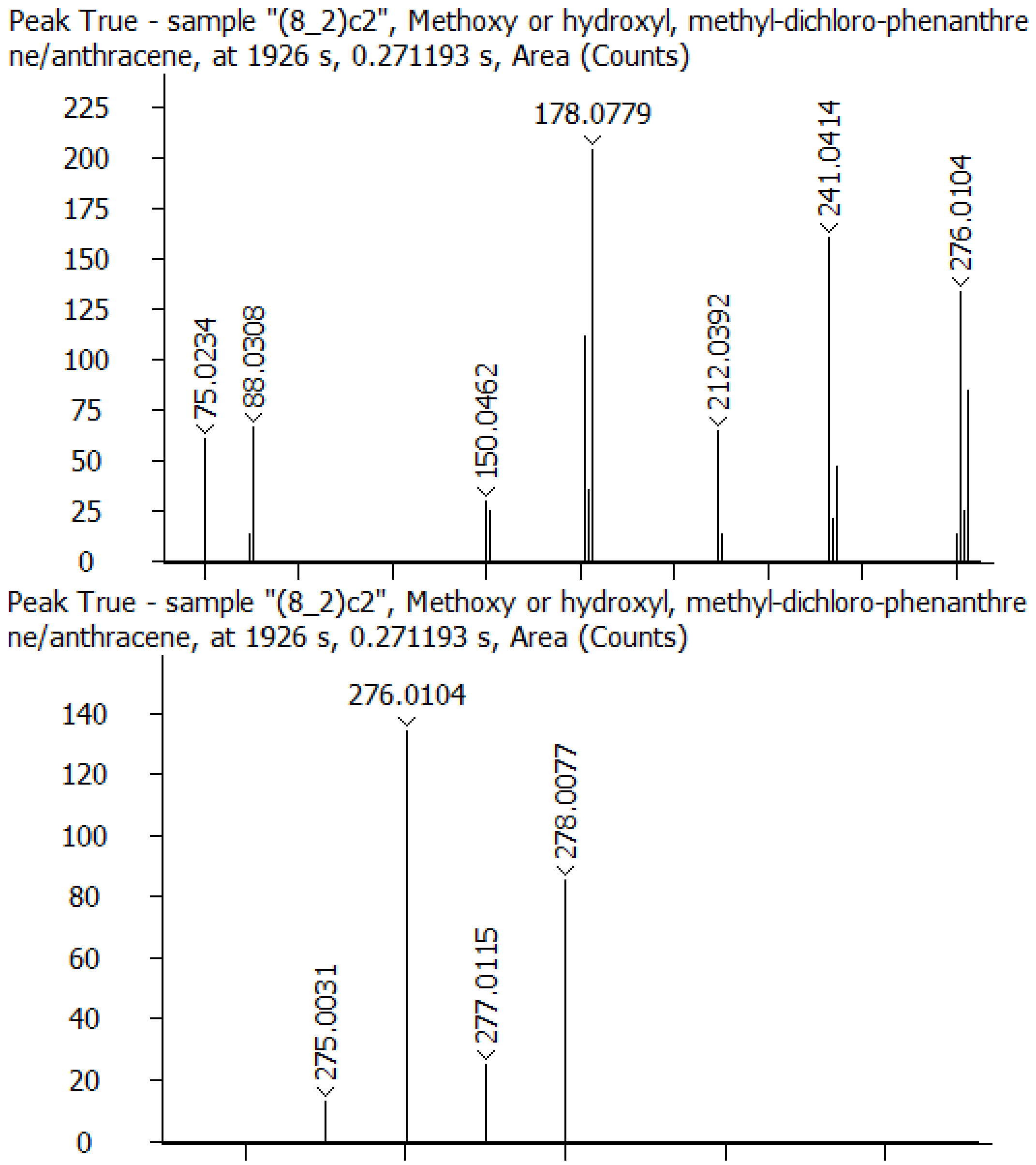

Figure S3 *Peak " Methoxy or hydroxyl, methyl-dichloro-phenanthrene/anthracene ", RT 1 (s): 1926, RT 2 (s): 4.27; top: whole spectrum, bottom: highest mass fragment*

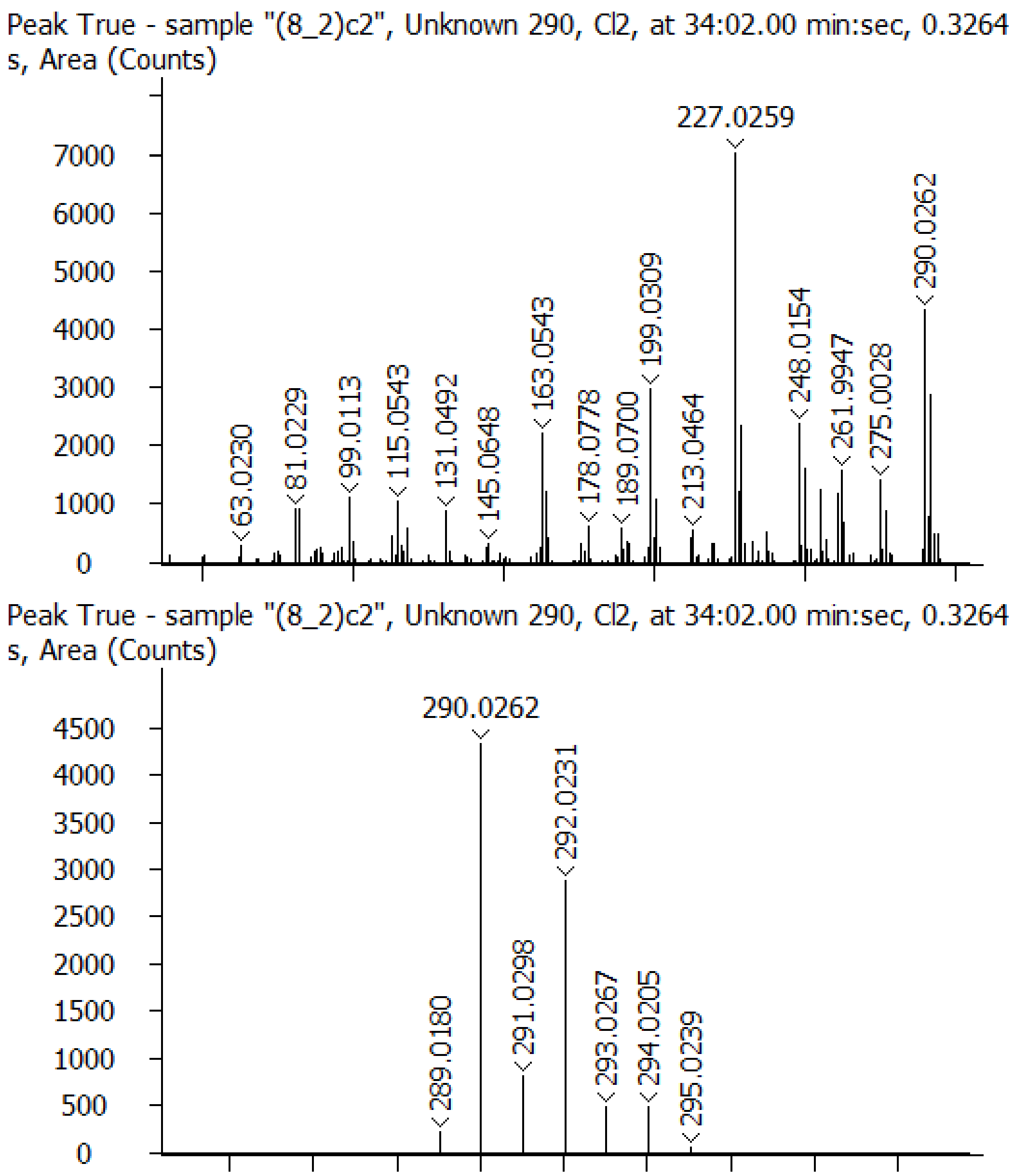

Figure S4 *Peak "4-(3,4-Dichlorophenyl)tetralone", RT 1 (s): 2042, RT 2 (s): 4.33; top: whole spectrum, bottom: highest mass fragment*

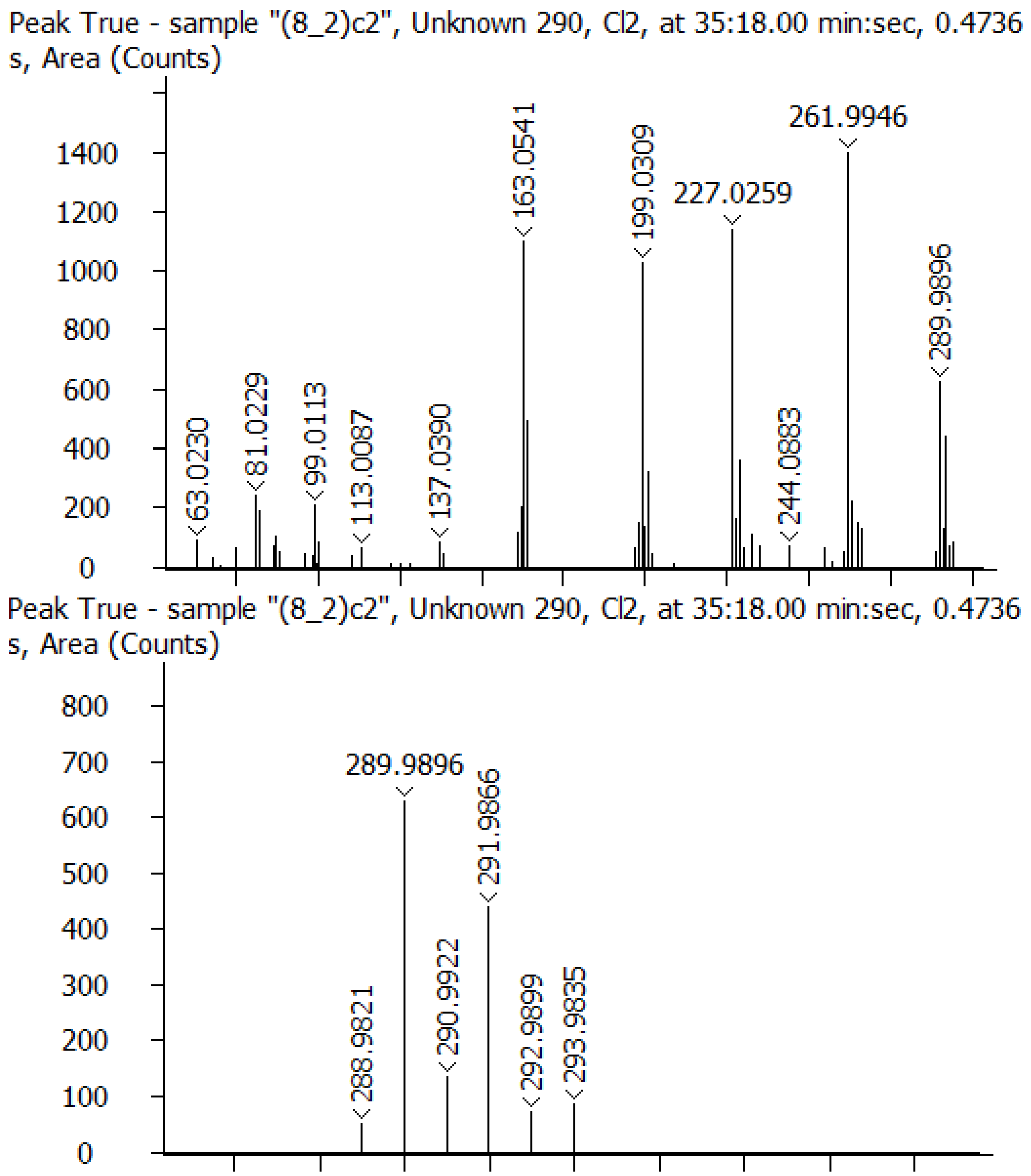

Figure S5 *Peak "Dichloroflavone or dichlorophenylcoumarin", RT 1 (s): 2118, RT 2 (s): 4.47; top: whole spectrum, bottom: highest mass fragment*

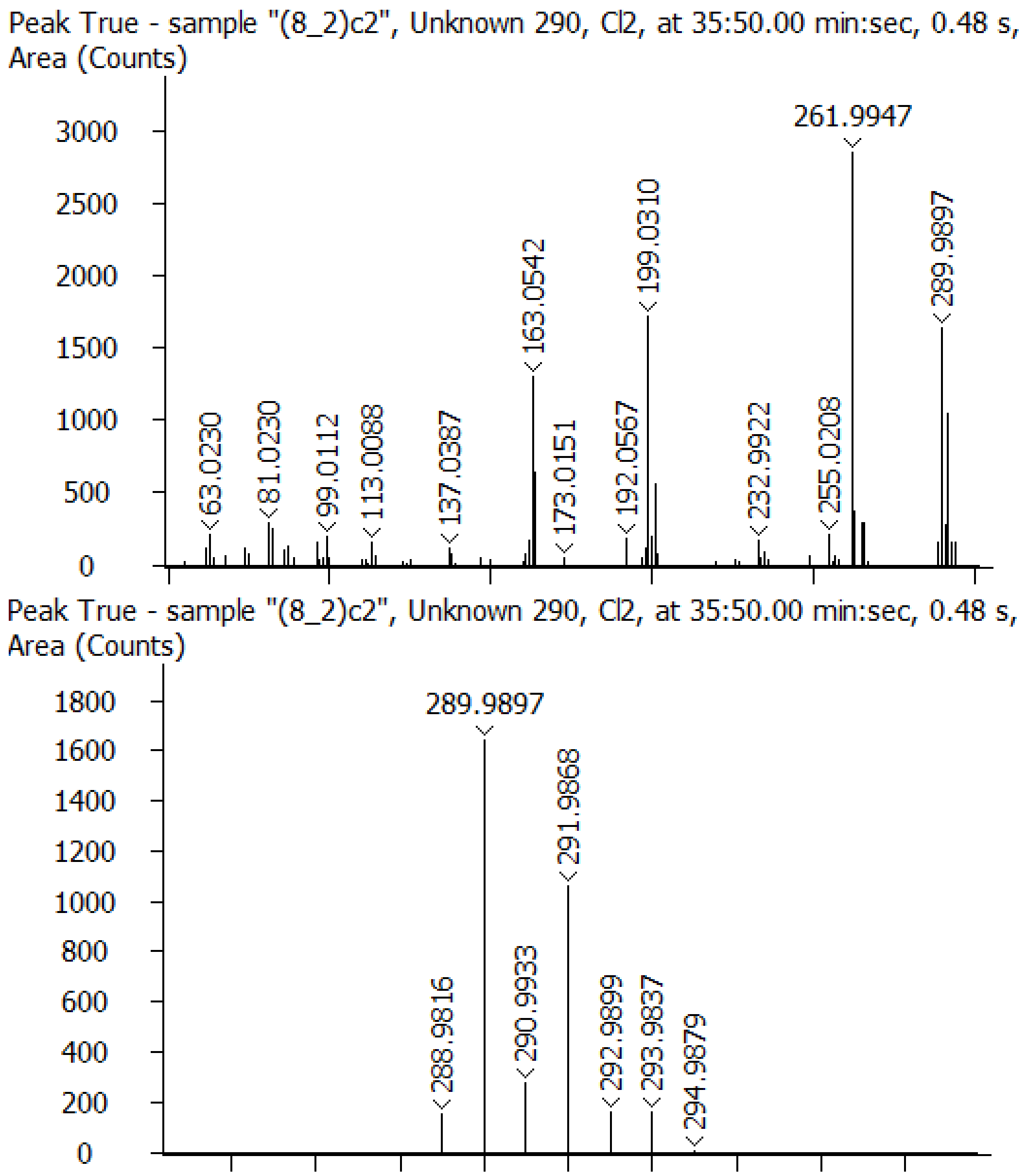

Figure S6 *Peak "Dichloroflavone or dichlorophenylcoumarin", RT 1 (s): 2150, RT 2 (s): 4.48; top: whole spectrum, bottom: highest mass fragment*

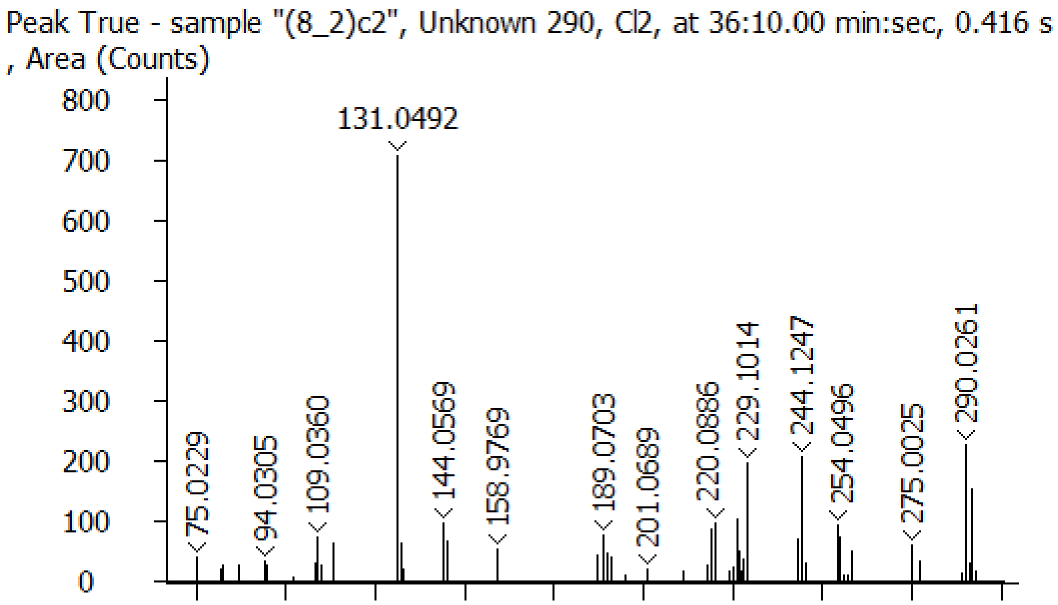

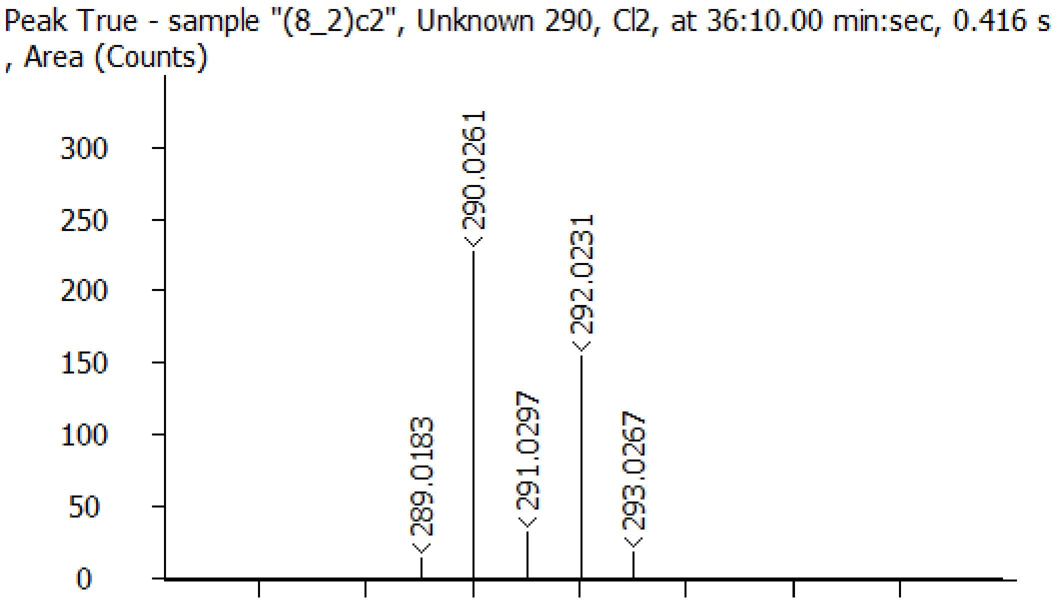

Figure S7 *Peak "Isomer of 4-(3,4-dichlorophenyl)tetralone", RT 1 (s): 2170, RT 2 (s): 4.42; top: whole spectrum, bottom: highest mass fragment*

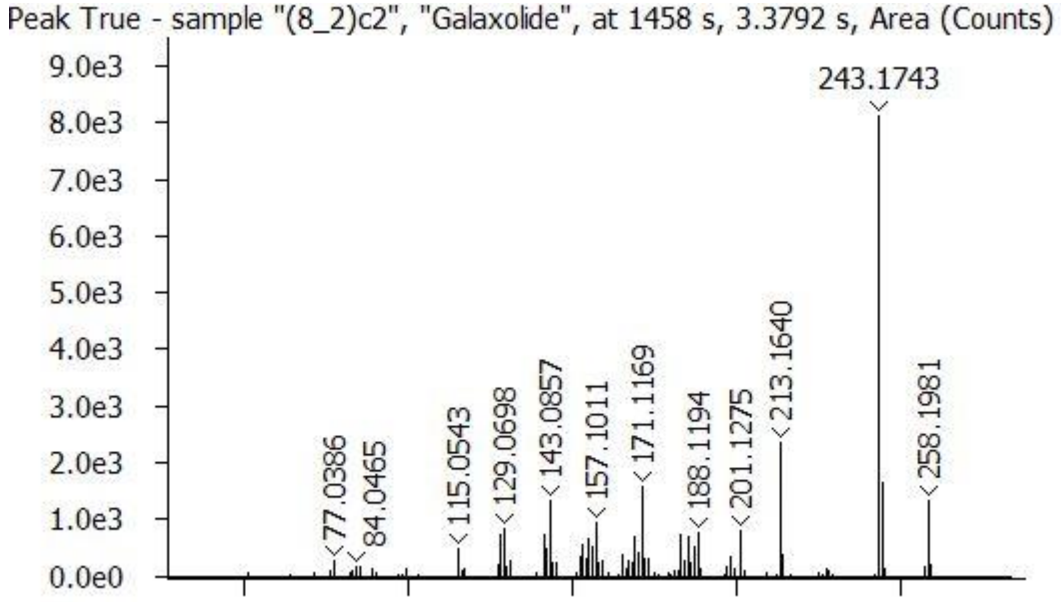

Figure S8 *Peak "Galaxolide impurity (1)", Rt 1 (s): 1458, Rt 2 (s): 3.38*

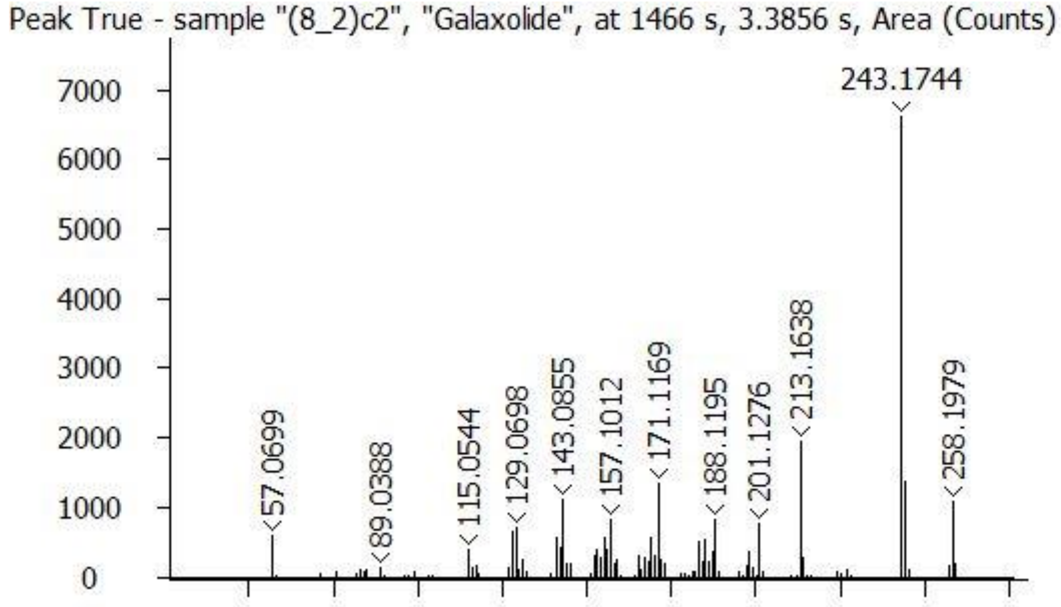

Figure S9 *Peak "Galaxolide impurity (2)", RT 1 (s): 1466, RT 2 (s): 3.39*

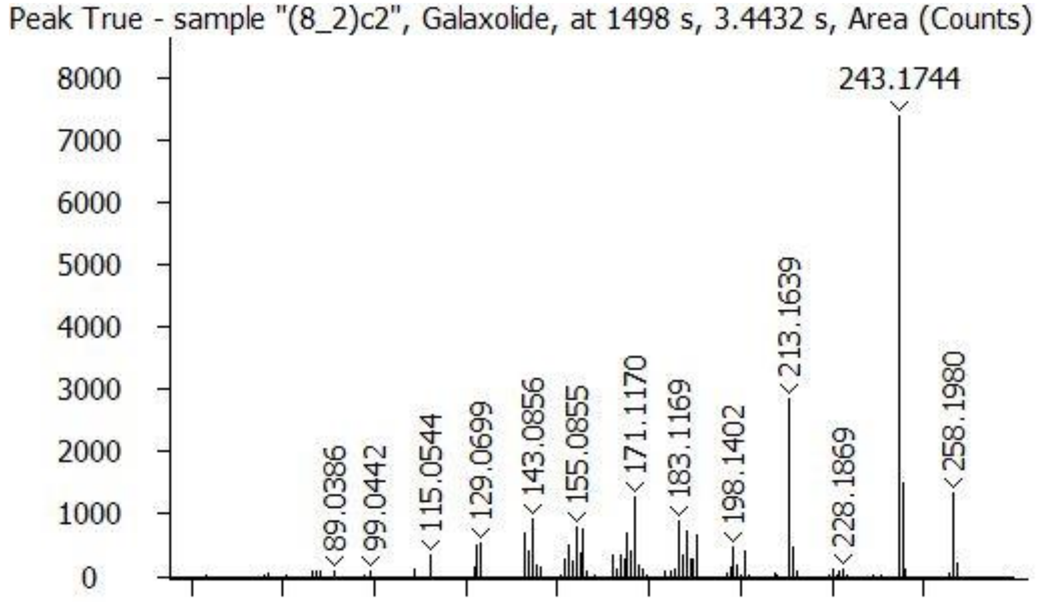

Figure S10 *Peak "Galaxolide impurity (3)", RT 1 (s): 1498, RT 2 (s): 3.44*

References

1. Sprecker MA (1987) Single phase liquid mixture of tricyclic isochroman derivative mixture and acetyl tetrahydronaphthalene derivative mixture. U.S. Patent 4,650,603.
